# The Confluence of Nanotechnology and Heat Shock Protein 70 in Pioneering Glioblastoma Multiforme Therapy: Forging Pathways Towards Precision Targeting and Transformation

**DOI:** 10.1155/adpp/1847197

**Published:** 2025-04-24

**Authors:** Amrita Arup Roy, Abhijeet Pandey, Namdev Dhas, Manasa Manjunath Hegde, Harendra S. Parekh, Sai Balaji Andugulapati, Krishnadas Nandakumar, Bola Sadashiva Satish Rao, Srinivas Mutalik

**Affiliations:** ^1^Department of Pharmaceutics, Manipal College of Pharmaceutical Sciences, Manipal Academy of Higher Education, Manipal 576104, Karnataka, India; ^2^Global Drug Development/Technical Research and Development, Novartis Healthcare Pvt. Ltd., Genome Valley, Hyderabad 500081, Telangana, India; ^3^Department of Radiation Biology and Toxicology, Manipal School of Life Sciences, Manipal Academy of Higher Education, Manipal 576104, Karnataka, India; ^4^School of Pharmacy, Pharmacy Australia Centre of Excellence, The University of Queensland, Brisbane, Queensland 4072, Australia; ^5^Department of Applied Biology, CSIR-Indian Institute of Chemical Technology (CSIR-IICT), Hyderabad 500007, Telangana, India; ^6^Department of Pharmacology, Manipal College of Pharmaceutical Sciences, Manipal Academy of Higher Education, Manipal 576104, Karnataka, India

**Keywords:** GBM, HSP70, nanotechnology, targeted therapy, therapeutic strategies

## Abstract

Heat-shock protein 70 (HSP70) and nanotechnology have emerged as promising avenues in glioblastoma multiforme (GBM) therapy, addressing the critical challenges posed by its aggressive nature and therapeutic resistance. HSP70's dual role in cellular stress response and tumour survival emphasises its potential as both a biomarker and therapeutic target. This review explores the innovative integration of HSP70 with nanotechnology, emphasising advancements in imaging, drug delivery and combination therapies. Nanoparticles, including SPIONs, liposomes, gold nanoparticles and metal–organic frameworks, demonstrate enhanced targeting and therapeutic efficacy through HSP70 modulation. Functionalized nanocarriers exploit HSP70's tumour-specific overexpression to improve drug delivery, minimise off-target effects and overcome the blood–brain barrier. Emerging strategies such as chemophototherapy, immunotherapy and photothermal therapy leverage HSP70's interactions within the tumour microenvironment, enabling synergistic treatment modalities. The review also highlights translational challenges, including heterogeneity of GBM, regulatory hurdles and variability in the enhanced permeability and retention (EPR) effect. Integrating computational modelling, personalised approaches and adaptive trial designs is crucial for clinical translation. By bridging nanotechnology and molecular biology, HSP70-targeted strategies hold transformative potential to redefine GBM diagnosis and treatment, offering hope for improved survival and quality of life.

**Trial Registration:** ClinicalTrials.gov identifier: NCT00054041 and NCT04628806

## 1. Introduction

The widespread threat that cancer poses to human health makes it a significant global issue for public health. Numerous diagnostic and treatment approaches have been suggested and implemented due to the significant investments made in cancer research in recent years. However, due to the distinct characteristics of malignant tumours, including their heterogeneity, immune-evasion capacity, infinite capacity for replication and capacity for metastasis, traditional cancer diagnostic and treatment approaches face significant challenges. Therefore, there is an increasing need to create new cancer detection and treatment techniques. Glioblastoma multiforme (GBM) is one of the highly aggressive brain tumours classified as WHO grade IV, which are most prevalent primary brain tumours, accounting for 81% of malignant brain tumours. Glioblastoma is a common type of glioma, accounting for around 45% of all gliomas. These tumours are highly aggressive and cause significant mortality and morbidity. The 5-year relative survival rate for glioblastoma is only 5%, highlighting its deadly nature and limited treatment success [1].

The annual incidence of primary malignant brain tumours is approximately about 7 per 100,000 people and has the lowest survival rate [2]. The chances of developing GBM increase with age, with the highest occurrence observed in the 75- to 84-year-old age group in the United States [3]. It is more commonly observed in men than in women and has a higher prevalence among Caucasians compared to other ethnicities [4].

Research utilising gene profiling and genetic modelling in mice suggests that GBM originates from oligodendrocyte precursor cells (OPCs), NSC-derived astrocytes and neural stem cells (NSCs). GBM tumours arising from different cellular origins exhibit distinct behaviours in animal models [5]. Despite the limited availability of drug therapies for glioblastoma, researchers are exploring innovative approaches to enhance pharmacological treatment. One promising strategy involves using nanomedicines, which offer efficient drug delivery systems. Various biocompatible nanodrug carriers, such as silica nanoparticles, nanoliposomes, magnetic nanoparticles (MNPs) and gold nanoparticles, have garnered attention in cancer research. However, achieving successful drug delivery requires nanoparticles that meet specific requirements. To address these challenges, scientists are continuously designing and optimising nanocarriers to improve the efficacy of glioblastoma treatment [6]. One of the major drawbacks of glioblastoma is accurate targeting and therefore a need for accurate biomarkers. HSP70 is highly expressed in GBM and is often associated with poor prognosis, highlighting its significance as a potential biomarker and as a therapeutic target for the disease [7].

### 1.1. Heat-shock Proteins (HSPs)

In 1962, Ritossa discovered that heat has an impact on the chromosome puffing patterns of *Drosophila*. This discovery led to the identification of heat-inducible genes and proteins and opened new avenues for research on the heat shock response. It was later discovered that high expression of HSPs, especially HSP70, plays a protective role against cellular stress [8]. Further investigations revealed that HSP70 functions as a molecular chaperone, facilitating cell survival by catalysing the reassembly of damaged ribonucleoproteins [9]. HSP70 performs various housekeeping and stress-related functions, including protein folding, protein degradation, prevention of protein aggregation, its transport across the membranes, and assembly/disassembly of protein complexes [10].

The HSP70 protein family, which has multiple homologues in prokaryotes and eukaryotes, is highly conserved throughout evolution [11]. In humans, there are 13 HSP70 homologues located in different cellular compartments, indicating distinct biological roles specific to each compartment [10].

HSP70 proteins consist of three main domains:

1. N-terminal ATPase domain: Binds ATP and hydrolyses it to ADP, leading to conformational changes.
2. Substrate-binding domain: Contains a groove that binds hydrophobic peptides up to seven residues in length.
3. C-terminal domain: Acts as a lid for the substrate-binding domain, controlling peptide binding and release.

**Figure 1:**
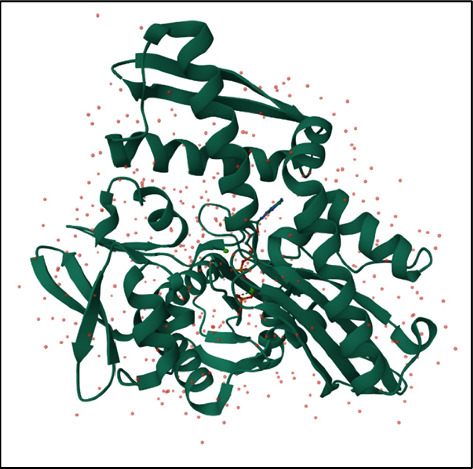
Structure of human HSP70 ATPase domain, PDB ID: 1S3X [12].

These domains work together to facilitate the chaperone function of the HSP70 proteins in protein folding and quality control processes. The image of the HSP70 protein is depicted in Figure 1.

HSP70, a highly conserved molecular chaperone, is central to various cellular processes, including protein folding, modulation of protein–protein interactions and degradation of damaged proteins. Its importance in maintaining protein homeostasis has made it a subject of intense study, particularly in the context of cancer.

**Figure 2:**
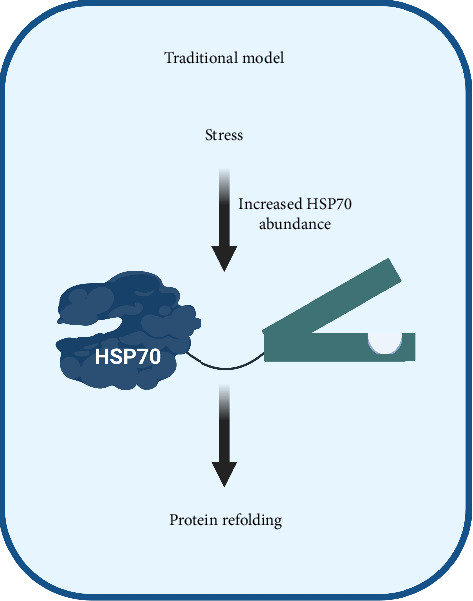
Cell stress induces protein unfolding, prompting increased HSP70 expression, which refolds proteins to restore cellular homeostasis. Adapted with permission from [13].

One of the classic modes of HSP70 regulation involves changes in its expression under stress conditions, tightly controlled by the master transcription factor HSF1. However, recent research has unveiled another layer of regulation through HSP70 phosphorylation. This post-translational modification, termed the ‘chaperone code', may intricately modulate HSP70's functions, adding a new dimension to its regulatory mechanisms (Figure 2).

**Figure 3:**
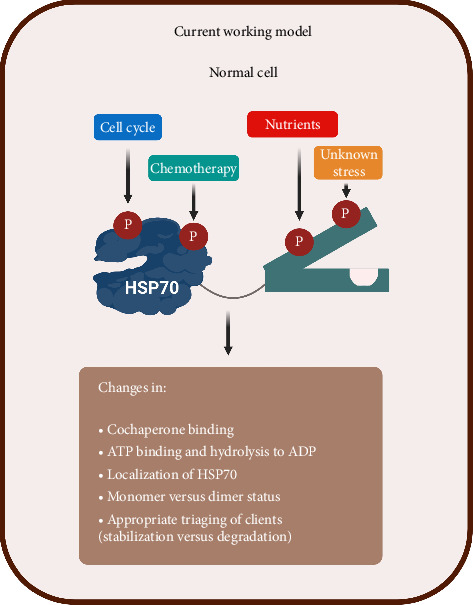
HSP70 regulation via phosphorylation: Various stimuli such as cell cycle progression, drug exposure and nutrient availability alter HSP70 phosphorylation in normal cells. Adapted with permission from [13].

Advances in phosphoproteomics, especially through mass spectrometry, have enabled the identification of numerous phosphorylation sites on HSP70. These sites, distributed across different regions of the protein, are now being scrutinised to understand their functional significance. Bioinformatics tools have been employed to prioritise these phosphorylation sites, shedding light on potential hotspots that may govern key aspects of HSP70 activity (Figure 3).

**Figure 4:**
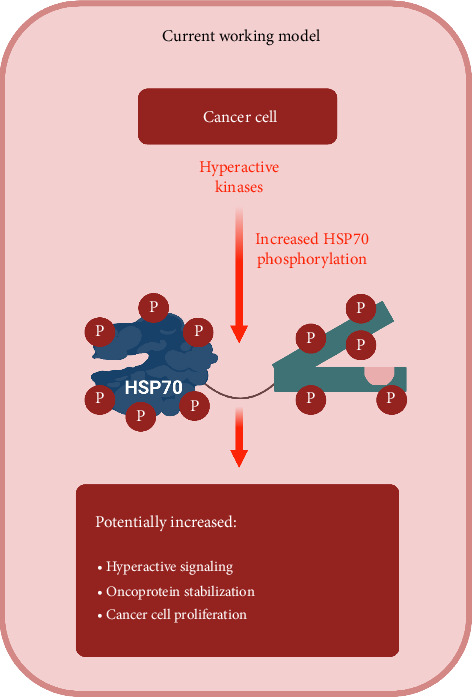
Misregulation of kinase activity due to random mutation in cancer cells. Adapted with permission from [13].

In cancer research, HSP70 has emerged as a critical player due to its involvement in promoting cell survival, inhibiting apoptosis and supporting protein homeostasis in tumour cells. Therefore, understanding the intricate regulatory mechanisms controlling HSP70 function, including phosphorylation, holds immense potential for developing targeted therapies against cancer. Furthermore, studies manipulating HSP70 phosphorylation sites have revealed their impact on cellular thermotolerance and protein binding, emphasising their functional relevance. This underscores the importance of unravelling the molecular intricacies of HSP70 regulation, particularly in the context of cancer, where dysregulated protein homeostasis contributes to disease progression. Experimental evidence shows high expression of HSP70 in malignant tumours, where it serves as a biomarker for poor prognosis (Figure 4) [14, 15].

This review focuses on the structure and the functional cycle of HSP70, its pathway to the extracellular environment and emerging research on its diverse role in cancer, relating them to the established Hanahan and Weinberg model of cancer hallmarks [16]. Understanding flexibility of the HSP70 machinery is crucial for the development of efficient anticancer therapeutics.

## 2. Pathophysiology of Glioblastoma

**Figure 5:**
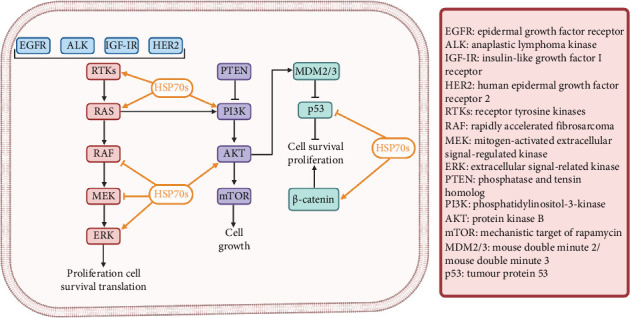
Schematic representation of cancer signalling pathways regulated by HSP70s. Adapted with permission from [17].

Glioblastoma involves complex cellular and molecular mechanisms contributing to its aggressiveness, rapid progression and resistance to treatments. Dysregulated signalling pathways, genetic mutations (e.g., EGFR, PTEN, TP53) and a complex tumour environment with invasive tendencies and angiogenesis contribute to its challenging nature which is schematically represented in Figure 5. Despite intensive efforts, treatment resistance persists due to factors like intratumoural heterogeneity and cancer stem cells (CSCs). Understanding and targeting these intricate mechanisms are crucial for developing more effective treatments against this formidable brain tumour.

### 2.1. Pathways in Glioblastoma

i. EGFR Pathway: The epidermal growth factor receptor (EGFR) pathway is frequently altered in GBM due to mutations and amplifications, leading to constitutive activation of signalling pathways driving cell proliferation and survival. The interaction between EGFR and HSP70 in glioblastoma is complex and influential, offering therapeutic opportunities alongside challenges. HSP70 binds to EGFR, preventing its degradation, enhancing signalling and contributing to treatment resistance. Cochaperones like CHIP and interactions with other pathways further impact EGFR activity. Targeting HSP70 could sensitise GBM cells to EGFR inhibitors, overcoming resistance. Strategies include dual inhibitors or leveraging HSP70 for drug delivery. However, developing specific HSP70 inhibitors without off-target effects remains a challenge, necessitating personalised approaches to address GBM tumour heterogeneity [17, 18].
ii. PI3K/Akt/mTOR Pathway: EGFR signalling activates the PI3K/Akt/mTOR pathway, promoting cell growth and inhibiting apoptosis. Dysregulation of this pathway is common in GBM, driving tumour progression. The relationship between HSP70 and PI3K/Akt/mTOR in cancer involves mutual activation and feedback loops. HSP70 also modulates mTOR activity and localisation. Targeting HSP70 to enhance sensitivity to PI3K/Akt/mTOR inhibitors offers potential for overcoming drug resistance. Challenges include understanding this complex network, developing specific inhibitors and addressing tumour heterogeneity. Further research is needed to leverage this interplay for precise and effective cancer treatments [19, 20].
iii. Ras/Raf/MEK/ERK Pathway: Aberrations in the Ras/Raf/MEK/ERK signalling cascade, which regulates cell growth and differentiation, are common in GBM, driving uncontrolled proliferation. HSP70 stabilises Ras, prolonging its signalling, and promotes Raf activation, facilitating ERK signalling. ERK, in turn, enhances HSP70 stability via phosphorylation, fostering cell survival. This reciprocal regulation underscores their intricate interplay in cancer. Targeting HSP70 to sensitise GBM cells to Ras/Raf/MEK/ERK inhibitors holds promise for overcoming resistance. However, challenges include unravelling this complex network, developing specific inhibitors and addressing tumour heterogeneity. Understanding these interactions could enable innovative cancer therapies [21].
iv. p53 and Rb Pathways: Dysfunction in the p53 and retinoblastoma (Rb) pathways disrupts cell cycle regulation and promotes unchecked cell division, contributing to tumour growth in GBM. The dynamic interactions between HSP70 and key tumour suppressors like p53 and Rb in cancer present both positive and negative impacts on cell fate. HSP70's stabilisation of wild-type p53 enhances its regulatory function, potentially prompting cell cycle arrest or apoptosis. Conversely, it can interfere with mutated p53, contributing to tumourigenesis. Additionally, HSP70's protective role in preserving Rb's cell cycle inhibitory function can be disrupted under certain stress conditions, leading to cell cycle progression. Leveraging HSP70 modulation as a therapeutic strategy presents challenges due to its multifaceted nature and potential off-target effects. Yet, understanding these intricate interactions offers promise for developing tailored interventions that capitalise on tumour suppressive pathways while navigating cancer's heterogeneity. This field remains ripe for further exploration, holding significant potential for innovative cancer therapies [22, 23].

### 2.2. Genetic and Molecular Alterations

Glioblastoma is characterised by genetic mutations and alterations in critical signalling pathways governing cell growth, proliferation and death. Genetic aberrations in genes such as EGFR, PTEN (phosphatase and tensin homolog), TP53 (tumour protein 53) and IDH (isocitrate dehydrogenase) are commonly observed. These mutations disrupt normal cellular functions, leading to uncontrolled cell division and tumour growth. HSP70 can also inhibit PTEN, further enhancing cell proliferation and survival. This creates a double whammy, favouring tumour growth. Mutations in IDH can lead to increased oxidative stress, which can activate HSP70. This creates a vicious cycle, as activated HSP70 can promote cell survival and resistance to therapy, further fuelling tumour progression [18].

### 2.3. Tumour Microenvironment (TME)

Glioblastoma develops within a complex microenvironment of diverse cell types, extracellular matrix (ECM) components and signalling molecules. Interactions among tumour cells, immune cells, endothelial cells and brain structures drive tumour growth, invasion, immune response and therapy resistance. HSP70 plays a multifaceted role in this microenvironment. In glioblastoma cells, elevated HSP70 levels support survival, inhibit apoptosis by interacting with pro-apoptotic proteins like Bax and Apaf-1 and enhance invasion, migration and therapy resistance. In the immune milieu, HSP70 both suppresses cytotoxic T lymphocyte activity, aiding tumour evasion, and stimulates the immune system under certain conditions by presenting tumour antigens to APCs. HSP70 promotes angiogenesis in endothelial cells and modulates tumour cell adhesion and migration by interacting with ECM components like fibronectin and collagen. The intricate interactions between HSP70 and the glioblastoma microenvironment pose challenges but also offer therapeutic opportunities. Targeting HSP70 in tumour cells holds promise for sensitising them to therapy and boosting the antitumour immune response. Combining HSP70-targeted therapies with immune-stimulating approaches, such as checkpoint inhibitors, could enhance treatment efficacy. Additionally, integrating strategies that target endothelial cells or ECM alongside HSP70-directed therapies provides a comprehensive approach to combating glioblastoma [24].

### 2.4. Invasion and Angiogenesis

Glioblastoma cells possess invasive capabilities, infiltrating surrounding healthy brain tissue, which makes complete surgical removal challenging. Moreover, glioblastomas induce angiogenesis, generating abnormal blood vessels to ensure a constant supply of nutrients and oxygen for sustained growth. HSP70 plays a crucial role in various aspects of glioblastoma's aggressive behaviour. It actively influences glioblastoma invasion by engaging in multifaceted processes. Firstly, HSP70 interacts with cytoskeletal proteins like actin and tubulin, fostering cellular protrusions and facilitating cell movement, thus promoting cell migration and motility. Additionally, HSP70 shields glioblastoma cells from apoptosis during invasion, ensuring their survival in challenging environments. Moreover, in the realm of glioblastoma angiogenesis, HSP70 proves influential. It stimulates proliferation of endothelial cells, crucial for the formation of new blood vessels. Additionally, HSP70 induces the secretion of pro-angiogenic factors, including vascular endothelial growth factor (VEGF) and other molecules, thereby fostering the process of angiogenesis. Furthermore, by contributing to the stability of newly formed blood vessels, HSP70 ensures a sustained and reliable supply of nutrients and oxygen essential for tumour growth [25, 26].

### 2.5. Therapeutic Resistance

Glioblastoma's resistance to standard therapies arises from factors like the blood–brain barrier (BBB) limiting drug penetration, intratumoural heterogeneity, CSCs with self-renewal and treatment resistance capabilities, and survival-promoting molecular pathways. The standard treatment includes surgery, radiotherapy and temozolomide (TMZ), a DNA-alkylating agent. However, GBM evades TMZ toxicity through DNA repair via O6-methylguanine-DNA methyltransferase (MGMT), cellular plasticity and glioma stem cells (GSCs). Additional resistance mechanisms involve drug efflux via ATP-binding cassette transporters, apoptosis blockade, autophagy, intracellular signalling adaptations, epigenetic modifications and metabolic reprogramming. The TME components, including astrocytes, endothelial cells, immune cells, ECM rearrangements and hypoxia, further support GBM survival and therapy adaptation. Understanding and addressing these resistance mechanisms are critical to improving GBM outcomes [26, 27].

### 2.6. HSP70 in Glioblastoma

**Figure 6:**
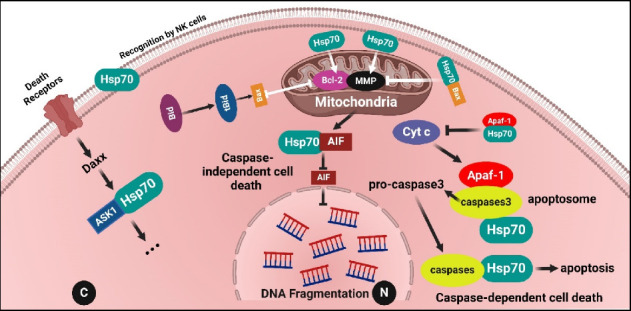
Schematic illustration showing the main pathways of apoptosis with the involvement of HSP70.

**Figure 7:**
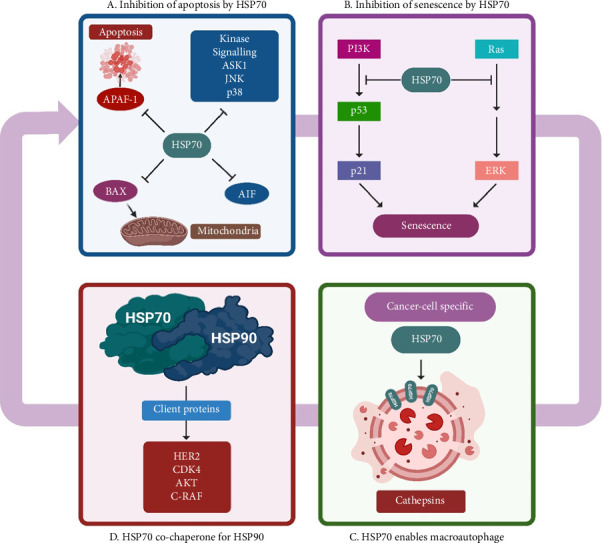
Role of HSP70 proteins in cancer development and regulation. Adapted with permission from [42].

HSP70, a member of the HSP family, plays a vital role as a molecular chaperone inside cells, aiding in proper protein folding, transportation and cellular protection during stress conditions [28]. In the context of glioblastoma:

i. Anti-apoptotic Effects: HSP70 has been implicated in inhibiting programmed cell death (apoptosis) by interfering with key apoptotic pathways. Elevated levels of HSP70 in glioblastoma cells may confer resistance to cell death induced by various therapeutic approaches.
  HSP70 plays a crucial role in apoptosis by blocking Bax, preventing mitochondrial membrane permeabilization and inhibiting the release of cytochrome c and AIF. This involves both the nucleotide-binding domain (NDB) and the chaperone function of HSP70 [29]. Additionally, HSP70 influences the expression of Bcl2 family members by inhibiting p53, which is often mutated in tumour cells, leading to the suppression of apoptosis [30]. Notably, overexpression of mutant p53 is associated with enhanced CSC properties through HSP70 upregulation. HSP70 emerges as a key player in regulating apoptosis and impacting tumourigenesis (Figure 6).
ii. CSCs: HSP70 plays a pivotal role in CSCs across various cancer types, contributing significantly to tumour progression and therapy resistance. Enhanced expression of HSP70 in CSCs is commonly observed and correlates with poor treatment response and increased aggressiveness, aiding in maintaining the CSC phenotype and promoting tumour advancement. Within CSCs, HSP70 regulates proteins associated with stemness, epithelial–mesenchymal transition (EMT) and resistance to cell death, crucial for sustaining CSC characteristics and survival. Its interaction with key signalling pathways like NFκB and c-Met fosters stemness, invasion and metastasis, contributing to tumour aggressiveness. Moreover, HSP70 confers resistance to stressors and therapeutic interventions in CSCs, shielding vital proteins necessary for their survival and treatment evasion. Extracellular and cell surface-bound HSP70 stimulates EMT and metastasis by activating pathways like p38/MAPK, driving CSCs towards a more invasive phenotype and facilitating metastatic spread [31].
  In a recent study focussing on HSP70's involvement in cancer metastasis and EMT, gene silencing techniques were used to create cancer cell lines with reduced HSP70 expression. These cells displayed characteristic EMT changes, including altered morphology, disrupted cell junctions, increased migration and enhanced sensitivity to anoikis. Immunofluorescence studies highlighted the impact of HSP70 loss, showing disorganised cell junction proteins like E-cadherin and β-catenin. This absence of HSP70 made cells more prone to anoikis and conferred mesenchymal traits and improved migratory capabilities. Previous hints about HSP70's involvement in EMT pathways were supported by this study, indicating that HSP70's presence delayed EMT by supporting cell adhesion. Conversely, its absence accelerated EMT and cell migration, suggesting potential therapeutic targeting against cancer metastasis [32].
  Targeting HSP70 in CSCs holds promise as a strategy to reduce stemness and sensitise them to therapies, with inhibitors of HSP70 function showing potential in combating CSC survival and improving treatment efficacy. Furthermore, surface-bound HSP70 on CSCs can be targeted using monoclonal antibodies, potentially inducing immunogenic cell death or inhibiting CSC-related activities, offering a promising avenue for CSC-specific therapy.
iii. Senescence: HSP70 plays a critical role in modulating the activity of p53, a pivotal tumour suppressor protein involved in regulating cellular responses to stress. By interacting with p53, HSP70 can inhibit its activation, thereby impairing its ability to induce cell cycle arrest, apoptosis or senescence in cancer cells [33]. This interaction is particularly significant in the context of HER2/neu-driven cancers, where p53-dependent senescence may occur as a response to oncogenic stress. However, the upregulation of HSP70 can counteract this process, allowing cancer cells to evade senescence induction and continue proliferating [34]. Therapeutically, targeting the interaction between HSP70 and p53 holds promise as a strategy to restore senescence induction in cancer cells, potentially inhibiting tumour growth. Disrupting this interaction could unleash the tumour-suppressive functions of p53, providing a novel avenue for cancer treatment, especially in HER2/neu-driven cancers where senescence evasion is a significant factor in tumour progression [35].
iv. Metastasis: HSP70 plays a critical role in promoting metastasis by aiding in tumour cell invasion and migration. It enhances the ability of cancer cells to break away from the primary tumour, invade surrounding tissues and enter the bloodstream or lymphatic system to reach distant organs. This process, known as metastasis, is a major contributor to the spread and progression of cancer throughout the body. HSP70's involvement in promoting tumour cell movement and invasion contributes to the aggressive nature of cancer and worsens patient prognosis [36].
  The mechanisms through which HSP70 promotes metastasis are multifaceted. Firstly, it stabilises proteins involved in cell adhesion, such as integrins and focal adhesion kinase (FAK), allowing cancer cells to interact with and penetrate the ECM more effectively. This increased adhesion and motility facilitate the invasion of cancer cells into surrounding tissues and blood vessels [37].
  Secondly, HSP70 enhances the activity of matrix metalloproteinases (MMPs), enzymes that degrade ECM components, promoting tumour cell migration through tissues. By facilitating ECM remodelling, HSP70 enables cancer cells to create paths for migration and invasion, further aiding in metastasis. Additionally, HSP70 contributes to the EMT, a process where cancer cells acquire traits that promote metastasis. It downregulates epithelial markers like E-cadherin while upregulating mesenchymal markers like N-cadherin and vimentin, leading to increased invasiveness and motility of cancer cells [38, 39].
v. Drug Resistance Mechanism: HSP70 is also intimately involved in drug resistance mechanisms in cancer cells. One of the primary mechanisms is its role in inhibiting apoptosis, or programmed cell death, which is triggered by chemotherapy drugs. High levels of cytosolic HSP70 prevent the activation of apoptotic pathways, allowing cancer cells to evade cell death induced by chemotherapeutic agents [36].
  HSP70 assists in folding and stabilising proteins involved in drug transport and metabolism. This chaperone activity can lead to the sequestration or inactivation of chemotherapy drugs within cancer cells, reducing their efficacy. Furthermore, HSP70 can induce resistance to HSP70 inhibitors themselves. This phenomenon, known as acquired resistance, limits the effectiveness of targeted therapies aimed at inhibiting HSP70's functions in cancer cells, leading to treatment failure and tumour recurrence [40].
  Understanding the intricate roles of HSP70 in promoting metastasis and drug resistance has significant clinical implications. Targeting HSP70 and its associated pathways could provide novel therapeutic strategies to combat metastatic disease and overcome drug resistance in cancer patients. However, developing required HSP70-targeted therapies requires further research to elucidate the specific molecular mechanisms and identify potential drug targets within the HSP70 regulatory network.
vi. Chaperone Function: HSP70's chaperone activity assists in protein folding, stability and degradation. In GBM, it may help in stabilising oncoproteins, aiding in accurate folding and function of mutated or misfolded proteins involved in oncogenic pathways.
vii. Autophagy: In cancer cells, HSP70 localises to lysosomal membranes, promoting cell survival by preventing lysosomal membrane permeabilisation and stabilising lysosomes [41]. This is crucial for autophagy, a survival pathway in cancer. Inhibiting HSP70, notably with PES, impairs autophagy, offering a potential therapeutic strategy to hinder cancer progression [21] (Figure 7).
viii. Promotion of Tumour Progression: HSP70 is involved in stabilising oncoproteins and signalling pathways related to cell survival and proliferation. Its increased expression in glioblastoma cells has been associated with enhanced tumour growth, aggressiveness and resistance to therapies.

### 2.7. Therapeutic Target Potential

**Figure 8:**
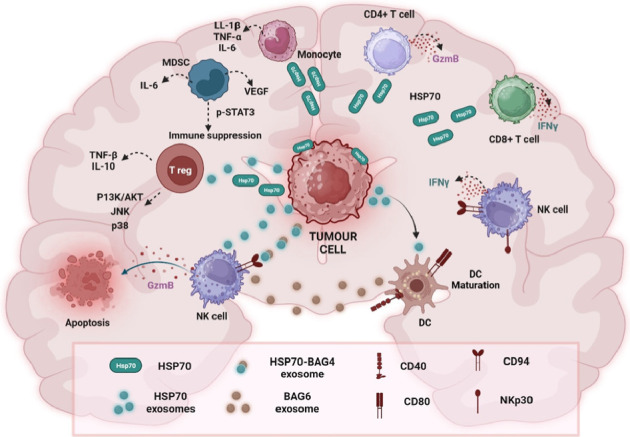
Extracellular HSP70 immune network in cancer. Adapted with permission from [46].

As research sheds light on the function of HSP70 in glioblastoma, it emerges as a potential therapeutic target. Strategies aimed at inhibiting HSP70 function or reducing its expression could sensitise tumour cells to apoptosis and potentially enhance the effectiveness of conventional glioblastoma treatments.

i. HSP70 Inhibition: Efforts are underway to develop small-molecule inhibitors that target HSP70's chaperone function, potentially sensitising GBM cells to apoptosis or making them more susceptible to other therapeutic interventions. VER-155008 is a small-molecule inhibitor of HSP70 that binds to its ATP-binding pocket, preventing its chaperone function. In preclinical studies, VER-155008 demonstrated efficacy in reducing glioblastoma cell viability and invasion. When combined with TMZ, a standard chemotherapeutic agent for glioblastoma, VER-155008 significantly enhanced tumour cell apoptosis and improved survival in animal models. This case study highlights the potential of HSP70 inhibitors to sensitise glioblastoma cells to chemotherapy and improve treatment outcomes [43].
ii. Antisense RNA: Silencing HSP70 through antisense RNA has demonstrated significant efficacy in inducing massive cell death in breast cancer (BCa) cell lines. This targeted approach suggests that selectively targeting HSP70 can trigger tumour cell death, offering a potential strategy for cancer treatment.
iii. Modulating the immune response: Extracellular HSP70 (eHSP70) displays a complex role in immune modulation, exerting dual effects in both immune activation and suppression, depending on interactions with diverse immune cells. It acts as a recognition factor for natural killer (NK) cells, enhancing proliferation, IFN-γ production, migration towards tumours and augmenting cytolytic activity [44, 45]. Engineered HSP70-expressing exosomes promote dendritic cell maturation, induce robust CD4+ and CD8+ T-cell responses and amplify cytokine secretion, fostering antitumour immunity (Figure 8). Conversely, eHSP70 contributes to immunosuppression by influencing myeloid-derived suppressor cells (MDSCs) through STAT3 phosphorylation, elevating IL-6 and VEGF secretion and modulating regulatory pathways in T regulatory cells (T regs) [47, 48].
  Additionally, surface GRP78/BiP on tumour and immune cells modulates various immune responses, impacting checkpoint regulation, cytokine secretion and signalling pathways [49]. Therapeutic strategies targeting eHSP70, such as specific antibodies against GRP78, exhibit promise in inducing apoptosis, complement-dependent cytotoxicity and restraining tumour growth across multiple cancer types [50]. Combinations of eHSP70-targeted therapies with strategies to enhance anti-tumour immune responses, like checkpoint inhibitors, show potential for improved efficacy. Moreover, studies have demonstrated that HSP70 inhibitors effectively reduce phospho-FAK levels in melanoma cells, inhibiting migration, invasion and metastasis in laboratory and animal models. Notably, inhibitors like PET-16 demonstrate efficacy in reducing mutant BRAF levels and act synergistically with BRAF inhibitors like PLX4032, potentially overcoming resistance encountered in melanoma patients undergoing BRAF inhibitor treatment. Understanding the intricate impact of eHSP70 on diverse immune components is critical for devising effective cancer treatment strategies targeting this molecule [51].
iv. Modulating pro-angiogenic factors: Blocking HSP70-mediated secretion of VEGF and other pro-angiogenic factors could starve tumour and limit its growth. HSP70 promotes the secretion of VEGF, a key pro-angiogenic factor. Researchers have developed small molecules that specifically inhibit HSP70-mediated VEGF secretion. In preclinical models, these molecules effectively reduced tumour growth and angiogenesis, demonstrating the potential of targeting HSP70–VEGF interaction for glioblastoma treatment [46].
v. Targeting cell adhesion and migration: Disrupting HSP70's interaction with cytoskeletal proteins or ECM components could hinder glioblastoma cell invasion. Integrins are cell surface receptors that mediate cell adhesion and migration. HSP70 interacts with integrins, promoting glioblastoma cell invasion. Researchers have developed peptide-based inhibitors that specifically disrupt HSP70–integrin interaction. These inhibitors effectively reduced glioblastoma cell migration and invasion in preclinical models, suggesting a potential therapeutic avenue for targeting cell motility [52].
  Through yeast two-hybrid screening, A8 and A17 peptide aptamers were identified to bind to different domains of HSP70, inhibiting its chaperone activity. When expressed in human tumour cells, these aptamers increased cell sensitivity to anticancer drugs, promoting apoptosis. A 13-amino acid peptide derived from A17, named P17, specifically inhibited HSP70 and caused regression of subcutaneous tumours in vivo. Notably, P17 treatment recruited macrophages and T lymphocytes to the tumour site. These observations highlight the potential of targeting HSP70 using peptide aptamers or derived peptides like P17 as a promising avenue for cancer therapy, offering novel strategies to combat cancer by inhibiting HSP70 and triggering antitumour immune responses [53].
vi. Metastasis Inhibition: HSP70 plays a pivotal role in promoting metastasis by enhancing tumour cell invasion and migration. Targeting HSP70 with specific inhibitors can disrupt these processes, thereby reducing the ability of cancer cells to spread to distant sites. This approach aims to block key signalling pathways involved in metastasis, such as p38/MAPK, which are activated by HSP70 [34, 52].
vii. HSP70 Co-chaperones: Modulating the function of HSP70 cochaperones like BAG3 and HOP can also impact tumour progression. For instance, knocking down HOP has been shown to inhibit pseudopodia formation and migration in BCa cells. Similarly, BAG3 plays a role in enabling MMP-2, which promotes invasion in ovarian cancer cells. Targeting these cochaperones alongside HSP70 presents an opportunity to disrupt critical pathways involved in cancer metastasis and invasion [53, 54].
viii. Overcoming Drug Resistance: High levels of HSP70 are associated with resistance to chemotherapy and other anticancer drugs. By targeting HSP70, either directly with inhibitors or indirectly through upstream regulators, it becomes possible to overcome drug resistance mechanisms. This can sensitise cancer cells to chemotherapy, leading to improved treatment outcomes and reduced risk of relapse [55].
ix. HSP70 as a Drug Target: Conjugating therapeutic agents or nanoparticles with molecules that specifically bind to overexpressed HSP70 on GBM cells is being explored. This approach aims to deliver cytotoxic drugs selectively to tumour cells while sparing normal tissues, potentially enhancing treatment efficacy and reducing side effects. Researchers are exploring the use of nanoparticles loaded with therapeutic agents conjugated with molecules that bind specifically to HSP70. This approach aims to deliver drugs selectively to glioblastoma cells overexpressing HSP70, minimising potential side effects on healthy tissues. In preclinical studies, HSP70-targeted nanoparticles showed promising results in delivering doxorubicin (DOX), a potent chemotherapeutic agent, to glioblastoma cells, leading to improved tumour cell killing and reduced systemic toxicity [56].
x. Combination Therapies: Combining HSP70-targeted therapies with conventional treatments like chemotherapy or immunotherapy can synergistically enhance their efficacy. For instance, using HSP70 inhibitors alongside chemotherapy can overcome drug resistance and reduce the metastatic potential of cancer cells. Similarly, combining HSP70 modulation with immunotherapy can boost antitumour immune responses, leading to better control of metastasis and reduced relapse rates [57].

Utilising HSP70 as a biomarker can aid in patient stratification and personalised treatment strategies. Patients with tumours showing high HSP70 expression may benefit more from HSP70-targeted therapies, guiding clinicians in selecting the most effective interventions to mitigate metastasis, drug resistance and relapse [58]. The development of monoclonal antibodies targeting mHSP70, which represents a high surface expression of HSP70 commonly found in metastatic tumours, presents a valuable opportunity. These antibodies can serve as biomarkers for assessing the metastatic potential of tumours, providing clinicians with crucial information to tailor treatment strategies and monitor disease progression effectively [59].

Understanding the complex interactions between HSP70 and the multifaceted pathophysiology of glioblastoma holds promise for developing novel therapeutic strategies. Targeting HSP70, along with comprehensive treatments addressing the diverse aspects of glioblastoma's molecular and cellular complexity, may offer new avenues for more effective management of this challenging disease. However, further investigation and preclinical studies are necessary to translate these findings into clinically viable therapeutic interventions.

## 3. Role of HSP70 in Glioblastoma

Various studies have proved that HSP70 is upregulated in numerous types of cancers, including glioblastoma, and is associated with disease progression and maintenance. In cases of primary GBM, it has been observed that not only is the cytosolic variant of HSP70 overexpressed but the membrane-bound 70 kDa heat shock protein (mHSP70) and secreted forms of the protein are also elevated. This suggests that HSP70 may have multifaceted roles in GBM, exerting its influence through different subcellular compartments [60].

In GBM, researchers have uncovered specific mechanisms by which HSP70 promotes cell survival and confers resistance to oxidative stress. The physical interaction between intracellular HSP70 and oxidised, inactive glyceraldehyde 3-phosphate dehydrogenase (GAPDH) in a rat model of GBM employing C6 cells was proven to be dose-dependent. This interaction, primarily mediated by HSP70's chaperone activity, prevents the aggregation of GAPDH. GAPDH is critical for the cellular response to oxidative stress; therefore, HSP70's restoration of its activity provides resistance and extends the survival of GBM cells [61].

Furthermore, recent advancements in super-resolution microscopy techniques have shed light on a unique function of mHSP70 in GBM. With stimulated emission depletion (STED) nanoscopy, it was discovered that mHSP70 participates in the clustering of tunnelling nanotubes (TNTs) in GBM cells. TNTs are membranous structures that facilitate cell-to-cell communication and transport of vesicles and organelles between cells. The presence of mHSP70, along with a lipid compound called globotriaosylceramide (GBM3), was found to be critical for the construction of TNT clusters in GBM tumour cells, highlighting the involvement of HSP70 in this essential cellular feature [62].

Glucose-regulated protein 78 (GRP78)/HSP5A, an endoplasmic reticulum-resident chaperone and a member of the HSP70 family, is known to be increased in several types of cancers, including GBM [63]. Targeting GRP78 interaction with various ligands has led to the identification of potential anticancer compounds [64]. For example, VH1019 and VH1011 have shown promising antiproliferative effects in various GBM cell lines, with specificity for tumour cells over healthy and normal nontumour cells [65].

Additionally, HSP70 plays a pivotal role in the maintenance of the stem cells in various brain tumours. Medulloblastoma-derived stem cells exhibit enhanced expression of HSP70, which activates the NF-κB signalling pathway through the regulation of its upstream client, Akt. This activation of NF-κB is critical for the proliferation and survival of medulloblastoma cells. In neuroblastoma models, HSP70 expression occurs during neuronal differentiation of precursor cells and is not associated with the proliferation status [66, 67].

**Figure 9:**
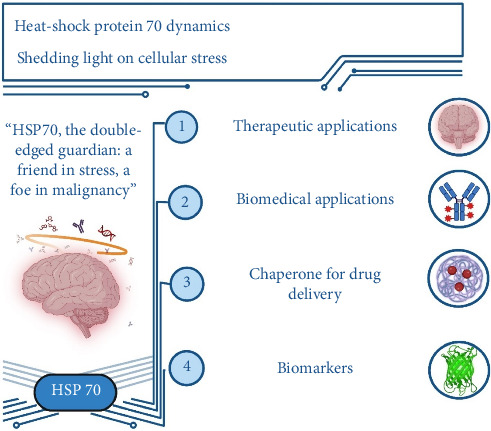
Role of HSP70 as a therapeutic and diagnostic target in glioblastoma multiforme.

Given the observed upregulation of HSPs in brain tumours and their involvement in glioma proliferation, apoptosis evasion and metastatic motility, HSPs have emerged as potential targets for the development of new therapeutic strategies against GBM as depicted in Figure 9. Active immunotherapy using HSP vaccines and target-based drugs are among the approaches being explored.

HSP vaccines involve isolating and purifying HSP–peptide complexes (HSPPCs) from resected GBM. These HSPPCs are then reintroduced into the patients to induce an immune response against tumour antigens. HSP vaccines can stimulate both innate and adaptive immune responses and have shown promising results in clinical trials [68]. The HSP–peptide complex-96 (HSPPC-96) vaccine has been extensively studied. It is comprised of autologous antigenic peptides chaperoned by HSP glycoprotein 96. Clinical trials using HSPPC-96 have demonstrated safety, minimal toxicity and the ability to elicit tumour antigen-specific immune responses in patients with recurrent GBM. Some trials have shown a correlation between vaccine-induced immune responses and increased overall survival in GBM patients [69, 70]. However, one of the major challenges in utilising HSP vaccines against the GBM is obtaining sufficient quantities of purified HSP-bound peptides for vaccine formulation manufacturing [71]. This hurdle needs to be overcome for the broader application and effectiveness of HSP-based immunotherapy in the treatment of GBM. Nonetheless, the potential of HSP70 as a therapeutic target in GBM and other cancers holds promise and warrants further investigation.

## 4. Challenges in Clinical Management of GBM

**Figure 10:**
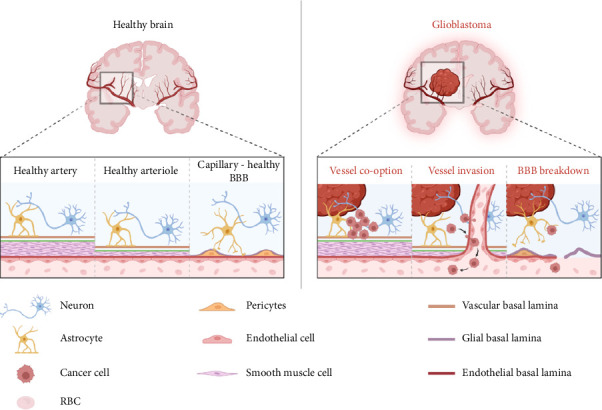
Healthy brain vasculature (left): normal endothelial cells, smooth muscle and pericytes in arteries, arterioles and capillaries. Perivascular space connects basement membranes to astrocytes. Molecules move through capillaries. GBM-affected vasculature (right): GBM invades along vessels, altering astrocytes and pericytes, forming niches for cell evasion.

Challenges in developing effective chemotherapy for glioblastoma include the BBB, tumour heterogeneity, drug resistance and limited survival benefits. The BBB's complex structure and active transporters impede drug transport [72–74]. In GBM, the blood–brain tumour barrier (BBTB) becomes more permeable, allowing potential nanocarrier transfer and drug accumulation [75–77] (Figure 10). GSCs and CSCs are crucial targets due to their self-renewal and tumour-initiating capabilities. Targeting GSCs could improve GBM prognosis [78–80]. Hypoxia in GBM, mediated by hypoxia-inducible factors (HIFs), particularly HIF2, promotes tumour invasion and contributes to radioresistance [81]. Chemicals mimicking oxygen are explored as radiosensitisers to overcome this resistance. Nanotechnology is investigated to create nanosized platforms that penetrate the BBB, delivering high drug concentrations for improved radiation and chemotherapy effectiveness in malignant brain tumours [82].

## 5. Current Drug Delivery Strategies for Brain Tumours

Brain tumours are a challenging target for drug delivery due to the BBB, which limits the delivery of drugs to the brain. However, there are several current drug delivery strategies that have been developed to overcome this barrier and effectively deliver drugs to brain tumours.

In intracranial implantable drug delivery systems, devices are directly implanted into the brain to provide localised drug delivery to tumours. Various implantable systems are designed for continuous drug delivery within the brain, addressing diverse neurological conditions such as glioblastomas, medulloblastomas, epilepsy, schizophrenia, stroke and Alzheimer's diseases. These systems encompass solid implants, in situ-forming implants, in situ-forming microparticles, depot formulations, polymeric-lipid implants, sucrose acetate isobutyrate and N-stearoyl L-alanine methyl ester [83]. Gliadel wafers, which are polymer-based wafer implants containing the chemotherapeutic agent carmustine, are implanted into the resection cavity after surgical removal of the tumour, and they slowly release carmustine over the treatment period [84].

Convection-enhanced delivery (CED) involves the injection of drugs directly into the tumour using a catheter. The drugs are then delivered to the tumour by convection, which is the flow of fluid through the tissue. This technique can be applied to many different drug classes, such as conventional chemotherapeutics or brand-new, investigational targeted medicines [85]. Drugs have been delivered using CED is IL-13-PE38QQR, a recombinant immunotoxin that targets the IL-13 receptor, which is overexpressed on glioma cells. In a phase I clinical trial, IL-13-PE38QQR was delivered by CED to patients with recurrent malignant glioma, and the results showed that the treatment was safe and well tolerated [86].

Focussed ultrasound (FUS) can be used to temporarily disrupt the BBB, allowing drugs to enter the brain. Combining FUS and the delivery of microbubbles can successfully break down the BBB, allowing medications to cross the cerebrovascular and enter the brainstem [87, 88]. This technique is still in the experimental stage and requires further development.

In order to eradicate tumour cells in a targeted manner, immunotherapy tries to stimulate an immune response specific to tumour. Immunotherapy strategies, such as the use of the checkpoint inhibitors, chimaeric antigen receptor (CAR) T-cell therapy, viral vector treatments vaccine-based strategies and cytokine-based treatment, have all been investigated for the treatment of GBM [89]. EGFRvIII-targeted immunisation stimulates patient immune responses despite therapeutic TMZ-induced lymphopenia and destroys EGFRvIII-expressing tumour cells without inducing autoimmune [90].

**Figure 11:**
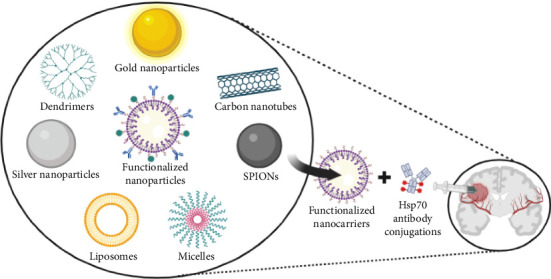
Functionalized nanoparticles explored as drug delivery systems for brain tumours.

Nanoparticles can be designed to cross the BBB and specifically target brain tumours as depicted in Figure 11. Different types of nanoparticles like gold, silver nanoparticles, SPIONs, dendrimers, MSNs, lipidic nanocarriers, etc. can be used to deliver chemotherapeutic drugs to the tumourous cells [91].

Elevated levels of HSP70 are often found in CSCs, contributing to their survival, proliferation and resistance to therapy. Nanoparticles can be designed to target HSP70-expressing CSCs specifically, utilising ligands or antibodies against HSP70 as targeting moieties on the nanoparticle surface. HSP70 plays a role in drug resistance mechanisms in CSCs, including efflux pump regulation and anti-apoptotic pathways. Nanoparticles can encapsulate inhibitors of HSP70 or other drugs that sensitise CSCs to chemotherapy, thereby overcoming HSP70-mediated resistance and improving treatment outcomes [92].

In addition to its intracellular functions, eHSP70 can modulate immune responses within the TME. Nanoparticles can be engineered to target eHSP70, either for inhibition to reduce immunosuppression or for stimulation to enhance antitumour immune responses against CSCs. Nanoparticles offer the advantage of delivering multiple therapeutic agents simultaneously. This can include HSP70 inhibitors, cytotoxic drugs, immunomodulators and imaging agents, creating a multifaceted approach to target CSCs and their microenvironment comprehensively. Nanoparticles with imaging capabilities can also be used to monitor HSP70 expression levels in real time within GBM tumours. This information can guide treatment decisions and assess the efficacy of HSP70-targeted therapies in reducing CSC populations [93, 94].

By incorporating HSP70 targeting into nanoparticle-based therapies for GBM CSCs, researchers can address the intrinsic characteristics of CSCs and the specific molecular pathways and resistance mechanisms associated with HSP70 expression. This integrative approach enhances the precision, effectiveness and therapeutic potential of nanoparticle-mediated treatments in combating GBM and improving patient outcomes.

Functionalized nanoparticles can be loaded with drug and delivered to the tumour. They can be loaded with drugs and functionalized with ligands to target specific receptors on the tumour cells. The potential for using nanocarriers as diagnostic tools is being examined in addition to their potential as a multipurpose platform for effective medication delivery [95, 96]. They have added advantages over other delivery systems such as enhanced drug accumulation, improved drug solubility, controlled drug release, ability to cross the BBB, which can be incorporated as combination therapy.

### 5.1. Nanoparticle Formulations Having Bound HSP70 for Treatment and Diagnosis of GBM

Nanocarriers are one of the most effective drug delivery systems that have received a lot of interest recently as a possible platform for targeted controlled drug delivery for different types of cancers, including brain cancer. Researchers have explored various nanocarrier systems to target and enhance the accumulation of cargo in tumour tissues. The strategy involves exploiting the leaky vasculatures of tumours, which permits nanoparticles to exit the vasculature and remain in the tumour tissues. The increased EPR effect is a tumour physiology-based phenomenon. Several factors play a pivotal role in higher-than-expected failure rates of phase II/III clinical trials with nanomedicines for cancer therapy. Recent studies of the preclinical data obtained from xenograft tumour models showed less than 2.25% injected nanoparticle-based dose accumulation in the tumour [97, 90]. Additionally, the EPR effect is known to be inconsistent and varies across and within individuals, which is one of the reasons phase II/III clinical studies employing nanomedicines for cancer therapy have shown higher-than-expected failure rates [98, 99]. Researchers have attempted to overcome these passive targeting challenges through the use of active targeting techniques, primarily antibodies or antibody fragments. Cellular internalisation can be significantly improved; however, antibody–nanoparticle conjugates would have to overcome the same delivery challenges that nontargeted nanoparticles face, such as accumulating in tumour tissue and travelling through tumour stroma, before targeting modality could enhance nanoparticle interaction with tumour and improve tumour specificity.

The 72 kDa heat-shock protein HSP70 (HSPA1A) has been identified on the membrane of various tumour cells, displaying a tumour-specific localisation absent in normal human cells. This specificity arises from the interaction between HSP70 and tumour-specific glycosphingolipids like globotriaosylceramide (Gb3), found in lipid rafts of tumour cells but lacking in normal cells. Under environmental stress, such as hypoxia, HSP70 associates with phosphatidylserine (PS) in tumour cells. Increased expression of mHSP70 is linked to heightened tumour aggressiveness and therapy resistance. Nevertheless, mHSP70 also serves as a valuable tumour-specific target for cancer diagnosis and therapy. Recent studies have successfully utilised fluorescence-, radionuclide- and MNP-labelled HSP70-targeting tools, including the full-length antibody cmHSP70.1, Fab fragment and tumour-penetrating peptide (TPP), for fluorescence, positron emission tomography (PET), computed tomography (CT) and magnetic resonance imaging (MRI) in preclinical models. In therapeutic applications, HSP70-targeting nanoparticles loaded with anti-survivin miRNA have demonstrated success in radiosensitising glioblastoma cells in vitro [100].

Nanoparticles, including SPIONs, gold nanoparticles, silver nanoparticles, quantum dots, selenium nanoparticles and metal–organic frameworks, synergise with HSP70-targeted strategies for enhanced cancer therapy. These nanomaterials, when conjugated or designed to target HSP70, exhibit selective tumour accumulation, improved imaging contrast and heightened therapeutic efficacy. From magnetic hyperthermia to chemotherapy, these nanoparticles coupled with HSP70 modulation demonstrate promising outcomes in glioblastoma, melanoma, BCa and other malignancies. Furthermore, dendrimers act as effective carriers for HSP70 inhibitors, and the theranostic nanocomplex ZIPP-Apt:DOX/siHSPs integrates magnetic hyperthermia and chemotherapy, showcasing the potential of combining nanotechnology and HSP70 targeting for advanced cancer treatments. The use of MOFs as a nanoenzyme for hypoxia modulation, guided by NIR-II PAI, highlights innovative approaches in addressing the challenges of the TME. This synergy between HSP70 and nanoparticles presents a multifaceted strategy for improving cancer diagnosis, treatment and monitoring as depicted in Table 1.

#### 5.1.1. Comparisons With Existing GBM Therapies

TMZ remains the cornerstone chemotherapeutic agent for GBM therapy. Acting as a DNA alkylating agent, TMZ induces cytotoxicity in proliferating tumour cells and is integral to the standard treatment protocol, often combined with radiotherapy [132]. Its oral bioavailability, predictable pharmacokinetics and ability to cross the BBB give TMZ a significant advantage. However, its clinical efficacy is limited by several factors, including resistance mechanisms driven by MGMT activity and dose-limiting systemic toxicities, particularly haematological side effects [133].

In contrast, HSP70-targeted nanotechnologies aim to address some of these limitations by leveraging the chaperone's role in tumour growth, apoptosis evasion and immune regulation. These nanocarriers offer enhanced specificity, reducing systemic toxicity and potentially improving therapeutic outcomes. Functionalized nanoparticles targeting HSP70 can exploit TME-specific features, such as high HSP70 expression, providing greater selectivity and reducing off-target effects [134, 135]. Additionally, their capacity for codelivery of chemotherapeutic agents and immunomodulators offers a multimodal strategy to overcome GBM's complex biology [136].

However, compared to TMZ, HSP70-targeted nanomedicines face significant translational challenges. TMZ's well-established mechanisms and predictable efficacy make it a reliable treatment option [137]. In contrast, nanotechnologies targeting HSP70 are still in experimental stages, with limited clinical data on their safety and efficacy [24]. Furthermore, while TMZ can efficiently cross the BBB due to its small molecular weight, most nanocarriers struggle with effective delivery to intracranial tumours, reducing their therapeutic impact [135].

#### 5.1.2. Challenges in Implementing HSP70-Targeted Nanotechnologies

Despite their potential, HSP70-targeted nanotechnologies face several hurdles that limit their clinical application.

#### 5.1.3. BBB Penetration

The BBB remains a major challenge for therapeutic delivery to GBM. While TMZ crosses the BBB effectively, nanocarriers often face significant limitations. Strategies such as surface functionalisation with ligands or receptor-mediated transcytosis have shown promise but remain inconsistent in clinical settings, resulting in reduced drug delivery efficiency [136].

#### 5.1.4. Heterogeneity of the TME

GBM's molecular and cellular heterogeneity complicates targeted therapies. Variations in HSP70 expression and the inconsistent EPR effect due to uneven tumour vascularisation can lead to suboptimal therapeutic outcomes [58, 59].

#### 5.1.5. Off-Target Effects and Toxicity

Although nanocarriers are designed for specificity, HSP70's expression in normal cells under stress raises concerns about unintended toxicity. Systemic delivery may lead to off-target effects, and the lack of extensive long-term toxicity studies further complicates safety evaluations [138].

#### 5.1.6. Resistance Mechanisms

GBM cells are adept at developing resistance through genetic mutations, epigenetic changes and compensatory survival pathways [139]. Since HSP70 supports protein homeostasis [140] and stress responses [141], targeting it could inadvertently trigger resistance mechanisms, reducing therapeutic efficacy [142].

#### 5.1.7. Integration With Current Therapies

TMZ and radiotherapy form the backbone of GBM treatment, with their combination efficacy well established. For HSP70-targeted nanocarriers to gain clinical acceptance, they must demonstrate superior efficacy and compatibility with these existing therapies [143]. Studies exploring synergistic effects between nanocarriers and conventional therapies are limited, delaying their integration into treatment protocols [144].

#### 5.1.8. Regulatory and Manufacturing Challenges

Nanomedicines face stringent regulatory requirements, including comprehensive characterisation, safety profiling and quality control. Scaling up the production of HSP70-targeted nanocarriers while maintaining consistent quality presents significant challenges. Regulatory guidelines vary globally, necessitating region-specific adaptations [145].

#### 5.1.9. Patient-Specific Variability

The clinical efficacy of HSP70-targeted nanotechnologies can vary significantly due to patient-specific factors, such as tumour genetics, immune status and prior treatments. Personalised approaches may enhance outcomes but add complexity to implementation in routine clinical practice [141].

To overcome these challenges, a multidisciplinary approach is essential. Advances in BBB penetration techniques, predictive computational modelling and TME targeting must be combined with robust preclinical and clinical studies. Collaborative efforts with regulatory agencies to streamline approval processes, adaptive trial designs and the exploration of synergistic combinations with existing therapies like TMZ will be pivotal. Successfully addressing these challenges will determine the clinical viability of HSP70-targeted nanotechnologies as a transformative option in GBM therapy.

### 5.2. Biomedical Application in Cancer

Depending on whether it is localised intracellularly or extracellularly, the important stress-inducible HSP70 performs several functions. HSP70 hinders pro-apoptotic signalling pathways in the cytosol, protecting tumour cells from programmed cell death. According to research publications, eHSP70 and proinflammatory cytokines promote the expression of activator NK cell receptors, which recognise exceptionally aggressive human tumour cells that possess HSP70 on their cell surface [146].

A previous study explored how HSP70 and activated NK cells could potentially predict prognosis in patients with glioblastoma. According to the study, gliomas all tested positive for membrane HSP70, with high-grade gliomas displaying a higher frequency of HSP70 overexpression in both the nucleus and cytoplasm. Additionally, elevated levels of eHSP70 were found in glioblastomas with significant necrotic areas. It has been found that low levels of HSP70 in the serum are associated with a better overall survival rate, indicating that high HSP70 expression is linked with an unfavourable prognosis [147].

Recent research published in Nature's Scientific Reports in 2021 discovered that extracellular vesicles containing HSP70 have the ability to trigger adaptive immunity in mouse models of melanoma and colon carcinoma. According to these studies, HSP70 has the potential to serve as a biomarker for predicting the prognosis of glioblastoma patients and may also be useful for immunotherapy. Extensive research is being done to fully understand the function of HSP70 in malignant cells and its potential in the aid of biomedical applications.

#### 5.2.1. Photothermic and Photodynamic Therapies

Research is being carried out focussed on the prospect of photothermal therapy (PTT) for cancer treatment, specifically mild PTT, which has lower side effects compared to high PTT. However, challenges such as inflammatory response, immune dysfunction and also limited penetration depth in biological tissues need to be addressed [148]. High PTT is hindered by poor penetration depth and potential side effects on surrounding tissues [149].

Researchers sought to combat thermotolerance brought on by the elevated expression of HSP in tumourous cells in order to increase the effectiveness of mild PTT. PTT and chemotherapy were combined in earlier research, but these methods had drawbacks and required particular circumstances. [150]. The effectiveness of mild PTT was shown to be improved by combining it with the HSP70 inhibitor VER-M. For mild PTT, they chose MPEG-AuNR as a model material. But study is also reported in which mild temperature PTT, with its safety and specificity, faces limitations due to HSP overexpression. To enhance therapeutic outcomes, liposome-templated gold nanoparticles were used for precise photothermal temperature control. HSP70 expression was minimised at 47°C, the optimal temperature for HSP management. Co-administration of an HSP70 inhibitor during 47°C PTT resulted in significantly enhanced tumour inhibition. This temperature-controlled PTT, guided by HSP70 expression, presents a novel strategy for improving clinical outcomes in cancer treatment [151].

Combining methoxy-polyethylene-glycol-coated-gold-nanorods (MPEG-AuNR) with VER-155008-micelles in PTT enhances heat sensitivity of tumour cells. VER-155008 micelles downregulate HSP70 expression, attenuating heat resistance in HCT116 cells. The system achieves significant tumour size reduction, demonstrating the potential of VER-155008-micelles to improve therapeutic outcomes in mild-temperature PTT. This approach highlights the relevance of HSP70 modulation in PTT for effective cancer treatment [152]. In another approach, combining mild-temperature PTT with glucose oxidase (GOx) and HSP inhibitor offers enhanced efficacy while minimising toxicity. Thermosensitive GOx/indocyanine green/gambogic acid (GA) liposomes (GOIGLs) synergistically inhibit tumour HSP through GA release and GOx-induced glucose consumption. This strategy of tumour starvation and phototherapy significantly enhances mild-temperature PTT efficiency. The approach demonstrates a delicate platform for combining PTT with other therapeutic methods, showcasing the potential for more efficient cancer treatment through the synergy between HSP70 and PTT [153].

Researchers have designed near-infrared (NIR-II) light excitation phototheranostics nanomedicine, Lip(PTQ/GA/AIPH), is developed for triple-negative breast cancer (TNBC). Integrating semiconducting polymer, azo compound and HSP inhibitor into a thermosensitive liposome, it offers tumour-targeted NIR-II fluorescence and photoacoustic dual-modal imaging, along with NIR-II PTT. The HSP inhibitor enhances PTT effectiveness by overcoming heat resistance. Azo compound, activated by NIR-II, generates cytotoxic free radicals for oxygen-independent photonic thermodynamic therapy (PTDT). This nanomedicine achieves precise diagnosis and effective TNBC suppression, presenting a promising strategy for HSP70-guided PTT in cancer treatment [154].

A composite hydrogel containing erastin and zinc-centred carbon nano-dodecahedrons (ZCND) was created for cancer treatment postsurgery. HSP70 is downregulated and ferroptosis effectiveness is increased by the hydrogel, which combines photodynamic therapy (PDT) and PTT. The method successfully prevents postsurgical cancer recurrence by effectively suppressing tumour growth after excision [155]. Photofrin-based PDT induces the surface expression and release of HSP70 in SCCVII tumour cells. This phenomenon occurs immediately after PDT treatment and persists for several hours. Similar outcomes were observed in the human umbilical vein endothelial cells. Higher PDT doses led to prompt HSP70 release, while lower doses induced release over time. When macrophages were exposed to PDT-treated tumour cells, they showed increased levels of HSP70 and GRP94 on their surface and released tumour necrosis factor A. Inhibition of HSP70, Toll-like receptors or NF-KB resulted in suppression of tumour necrosis factor A production. During PDT, the expression of HSPs on the surface and their release plays a significant part in the immune and inflammatory responses that aid in achieving a positive therapeutic result [156].

#### 5.2.2. Chemophototherapy

A zeolitic imidazolate framework-8 (ZIF-8) nanoplatform with chondroitin sulphate (CS) functionalisation, CS/ZIF-8@A780/DOX NPs, was designed for chemophototherapy for overcoming cancer therapeutic resistance. To overcome drug resistance and phototherapy tolerance, the nanoplatform combines atovaquone (A780) with DOX, both of which are conjugated with the photosensitiser IR780. This combination manipulates energy metabolism and bypasses drug efflux transporters and HSP70. P-glycoprotein (P-gp) and HSP70 activity are both suppressed as a result of ATO and zinc ions produced in the cancer microenvironment. With CS applied to its surface, the nanoplatform's targeting ability was also increased. This approach offered a potentially effective way to improve chemophototherapy and overcome cancer treatment resistance [157].

The nanoplatform, composed of biotin-tailored IR780 (B780) and quercetin (Qu), overcame the limitations of poor water solubility of both compounds. The B780/Qu nanoparticles (B780/Qu NPs) exhibited active tumour-targeting and pH-responsive drug delivery properties. The use of B780/Qu NPs in chemophototherapy resulted in remarkable effectiveness against tumours, while minimising side effects and suppressing the expression of HSP70. Tumour growth inhibition was significantly higher in the B780/Qu NPs + laser group compared to other groups. Moreover, the nanoplatform showed high biosafety and antitumour activity via chemophototherapy, as confirmed by tissue staining and analysis. This study provided a promising strategy for chemophototherapy and highlights the potential of targeting HSP-70 in cancer treatment [158].

Tan and his colleagues explored the efficacy of combined chemo-PTT using IR780-CSOSA/DOX micelles in MCF-7 tumour-bearing mice. The results demonstrated significant tumour volume reduction and scar recovery in the IR780-CSOSA/DOX group with laser irradiation. The therapy showed high antitumour efficiency and quite low systemic toxicity, with minimal organ damage and weight loss. Immunohistochemical analysis revealed increased HSP70 expression indicating local hyperthermia, and elevated cleaved caspase-3 levels indicating apoptosis. The combination therapy inhibited angiogenesis and activated the host immune response. Overall, this study presents a promising strategy for precise chemo-PTT with enhanced antitumour effects [159].

#### 5.2.3. Chemodynamic therapy (CDT)

Using a one-pot self-assembly technique, researchers developed FeEP-NP, a novel multifunctional nontherapeutic agent. This nanotherapeutic agent combines poly(vinylpyrrolidone) to function as stabiliser with FeII and (−)-epigallocatechin gallate (EGCG). Through the implementation of the Fenton reaction, FeEP-NPs effectively produce hydroxyl radicals (•OH) for CDT. Additionally, under NIR laser irradiation, mild hyperthermia-enhanced CDT is possible due to significant NIR absorption and photothermal conversion capabilities enabled by the binding between EGCG and FeII. The partially released EGCG elevates •OH production even more, increasing CDT, while further minimising the expression of heat-shock protein 90 (HSP90) to improve mild PTT. Studies conducted both in vitro and in vivo reveal that the low-temperature PTT-potentiated CDT based on FeEP-NP suppresses tumours more effectively. Future therapeutic applications hold considerable potential for this multifunctional nanoplatform due to its distinctive design [160].

Combining PTT along with CDT in one system has emerged as an attractive approach due to their synergistic mechanisms. Various nanomaterials have been developed that can stimulate photothermal conversion for PTT and release metal ions for CDT, providing simple distinctive platforms to integrate these modalities [161]. Hyperthermia from PTT can trigger drug release, enhancing the efficacy of Fenton-based agents. PTT not only leads to tumour cell death but also elevates the production of ROS and accelerates ROS oxidation, improving the efficiency of CDT. Additionally, ROS inhibition of HSPs enhances hyperthermia-induced tumour ablation [162].

#### 5.2.4. Sonodynamic Therapy (SDT)

In GBM, the combination of HSP70 with SDT can improve treatment by enhancing cytotoxic effects, sensitising cells to apoptosis, regulating the heat shock response, and overcoming therapeutic resistance. HSP70 can promote the accumulation of sonosensitiser chemicals, resulting in increased cytotoxicity following ultrasound activation. HSP70's anti-apoptotic actions can be reversed by targeting it, making GBM cells more susceptible to SDT-induced apoptosis. HSP70 modification can cause the heat shock response to be disrupted, making GBM cells more vulnerable to SDT-induced hyperthermia. Inhibiting HSP70 can also overcome GBM resistance to chemotherapy and radiation treatment. More research is needed to test this concept and investigate HSP70 as a chemotherapeutic target in GBM SDT [163, 164].

#### 5.2.5. Immunophototherapy

Immunophototherapy, a biomedical application for cancer treatment, combines immunotherapy and phototherapy to enhance therapeutic outcomes. This method utilises the immune system's strength to eliminate cancer cells, coupled with light-based methods to destroy tumours and stimulate immune reactions [165]. It is vital to comprehend the significance of HSP70 in the realm of immunophototherapy. Exposure of tumourous cells to photothermal agents, such as nanoparticles that convert light into heat, can stimulate cellular stress and lead to elevated levels of HSP70. The increased HSP70 expression is not only a result of thermal stress but also due to elevation of ROS levels generated during the photothermal process.

HSP70 has been shown to have dual functions in immunophototherapy. Firstly, it acts as a cytoprotective factor by promoting cell survival and preventing apoptosis in response to thermal stress. This can be advantageous for normal cells in the vicinity of the tumour, as it helps protect them from collateral damage during photothermal treatment. However, in cancer cells, the upregulation of HSP70 can also confer thermoresistance and hinder the efficacy of phototherapy. Therefore, strategies to inhibit or modulate HSP70 expression in cancer cells are being explored to enhance the effectiveness of immunophototherapy [166].

Additionally, HSP70 has immunomodulatory properties that can influence the immune response against cancer. HSP70 can function as a chaperone for tumour antigens, assisting in their presentation to immune cells and promoting antigen-specific immune responses [167]. It can also activate dendritic cells and enhance antigen cross-presentation, leading to the activation of cytotoxic T cells. Furthermore, HSP70 can promote the release of proinflammatory cytokines and chemokines, facilitating immune cell recruitment and activation within the TME [168]. By integrating immunophototherapy and considering the role of HSP70, researchers are aiming to improve cancer treatment outcomes.

The study introduces a novel sandwich ELISA method, compHSP70, designed to detect HSP70 in liquid biopsies of tumour patients. Unlike conventional assays, compHSP70 can identify both free and exosomal HSP70, crucial for tumour biomarker research. Two monoclonal antibodies (cmHSP70.1 and cmHSP70.2) target specific epitopes on HSP70, ensuring high sensitivity and reliability. The assay exhibits excellent precision, recovery rates and linearity, unaffected by donor age or food intake. Clinical evaluation demonstrates significantly elevated HSP70 levels in NSCLC and glioma patients compared to healthy controls. Moreover, HSP70 concentrations correlate with tumour stage and decrease following radiotherapy, suggesting potential as a predictive biomarker and therapeutic monitoring tool [169].

#### 5.2.6. Radiation Therapy

The correlation between the level of HSP70 and radiation therapy has been explored, particularly in the context of squamous cell carcinoma of the head and neck (SCCHN). Monitoring tumour response is done in a mouse model of SCCHN, sHSP70 plasma levels were monitored in tumours after irradiation with 30 Gy. A decrease in serum sHSP70 levels was linked to a positive reaction to radiation therapy. The decrease in sHSP70 levels after radiation therapy might serve as a predictive factor for the clinical outcome of radiotherapy in humans. Radiation therapy induces cell death in tumour tissues. Dying cells release sHSP70, which contributes to the detectable levels of sHSP70 in the serum or plasma. The increase in sHSP70 levels after radiation therapy might reflect the release of sHSP70 by dying tumour cells. These research findings suggest that sHSP70 levels could be utilised as a potential biomarker for monitoring tumour response and predicting the clinical outcome of radiation therapy in SCCHN [170].

In another study, researchers investigate the role of hepatic radiofrequency ablation (RFA) heating parameters and their activation of HSPs in modulating distant tumour growth. Lower RF heating doses resulted in larger distant tumours, while higher RF heating doses led to less distant tumour growth. Ki-67 and microvascular density correlated with tumour growth. Lower-dose hepatic RFA had more periablational HSP70 compared to higher dose RFA. Anti-HSP70 micellar quercetin (MicQ) blocked distant tumour growth for both lower- and higher-dose RFA. This research sheds light on the interplay between HSP70 and RFA, presenting potential implications for cancer treatment strategies [171].

#### 5.2.7. Drug Delivery

HSP70 is expressed in various tumour cells and has been incriminated in tumour progression and resistance to programmed cell death. It occurs in both intracellular and extracellular forms, with distinct roles and effects on cancer. Intracellular HSP70 has a cytoprotective role by suppressing apoptosis and lysosomal cell death, while eHSP70 can stimulate tumourigenesis along with angiogenesis. However, the roles of HSP70 can be contradictory, as intracellular the HSP70 can also promote apoptosis, while the membrane-associated/eHSP70 can elicit antitumour immune responses. Considering the dual functions of HSP70, it presents an intriguing potential as a ‘double agent' in targeted cancer therapies. Targeting HSP70 could hold promise as a therapeutic strategy for human malignancies. However, there are challenges associated with developing HSP70-targeted therapies.

To address the relation between HSP70 and drug delivery, further research is needed. Investigating the interaction between HSP70 and drug delivery systems could potentially enhance the efficacy of cancer treatments. This area of research could explore how HSP70 levels and functions within tumour cells influence the delivery and effectiveness of anticancer drugs [26]. For example, researchers found that small extracellular vesicles (sEVs) which is derived from the adriamycin (ADR)-resistant BCa cells could transmit resistance to the drug to sensitive cells by delivering HSP70 [172].

Another study revealed that the administration of HSP70 inhibitors to HCT116 cancer cells led to a distinct phenotype associated with HSP70 inhibition. This included the downregulation of client proteins, such as the glucocorticoid receptor (GR), immunophilins (FKBP51 and FKBP52), the protein kinase Akt-1 and the cochaperone CHIP, ultimately resulting in apoptosis of tumour cells [173]. Injectable, biocompatible as well as biodegradable semi-interpenetrating polymer networks of collagen (COLL) and low-molecular-weight hyaluronic acid (LMW-HA) designed with gelatine particles were applied by researchers to develop a novel brain HSP70 delivery system [174].

Inhibiting HSP70 and its interactions with essential cochaperones, such as J proteins, nucleotide exchange factors (NEFs) and proteins with tetratricopeptide repeat (TPR) domains, represents an alternative strategy for targeting HSP70. Typically, these cochaperones bind to HSP70, regulating its diverse cellular activities. The significance of complexes formed between HSP70 and cochaperones in profolding, protrafficking pathways and prodegradation has been well established. Therefore, a promising approach involves preventing the interaction of HSP70 and its cochaperones with other proteins, or targeting allosteric sites that often disrupt these interactions [175].

#### 5.2.8. Herbal Therapy

Research done by Chowdhuri and his colleagues examined the impact of Brahmi's bacosides (BBM) on stress response in rats. Administered orally for seven days at 20 and 40 mg/kg doses, BBM did not significantly affect HSP70 expression in studied brain regions but countered stress-induced HSP70 increase. SOD activity decreased in the hippocampus with the lower BBM dose and increased in other brain regions with the higher dose. BBM induced P450 enzymes across all brain areas, with mixed responses to stress post-BBM treatment. The research suggestions at BBM's potential in preparing the brain for stress by modulating stress-related proteins like HSP70 and antioxidant enzymes like SOD. While not directly focussing on cancer therapy, these findings suggest BBM's role in stress management, which may indirectly influence aspects of cancer therapy. Further study is needed to understand BBM's precise mechanism and its implications for health and disease [174].

The study examined daphnetin, extracted from Changbai daphne, for its protective effects against hydrogen peroxide (H_2_O_2_)-induced damage in nerve-like PC12 cells. Daphnetin, known for anti-inflammatory and antitumour traits, showed promise in preventing cell death caused by oxidative stress. PC12 cells exposed to H_2_O_2_ experienced decreased viability and apoptosis. Daphnetin pretreatment reversed these effects, significantly increasing cell survival in a dose-dependent manner. It reduced the cleavage of apoptotic proteins like PARP and caspase 3 and preserved normal nuclear structure. A significant finding was daphnetin's ability to boost HSP70 expression, a protective protein against various stresses. Daphnetin increased HSP70 levels in a dose- and time-dependent manner, peaking at 12 h. The involvement of ERK signalling in this upregulation was highlighted. While the study primarily focussed on nerve cell protection, it indirectly suggested a potential role of daphnetin in cancer therapy due to its antitumour properties. The study highlights daphnetin's potential as a neuroprotective agent against oxidative stress-induced cell death and hints at its possible role in cancer therapy, although more research is needed for clarity [176].

Polyphenols like hydroxytyrosol and curcumin activate Nrf2-mediated pathways, inducing HSP70 and other vitagenes, showcasing potential in cancer prevention. Herbal agents, exemplified by HT-rich olive pulp extract, offer innovative avenues for cancer prevention and therapeutic strategies, tapping into redox stress responses and autophagy mechanisms [177].

#### 5.2.9. Gene Therapy

The transfer of genetic material to cells, also known as gene therapy, has undergone extensive research as a potential treatment for numerous illnesses associated to cancer. HSP70-based gene therapy has shown promise in laboratory research [178]. Gene transfer into the central nervous system often involves the use of vectors such as herpes simplex virus (HSV), adeno-associated virus (AAV) and lentivirus. In the context of brain ischemia and related conditions, gene therapy with HSV overexpressing HSP70 has demonstrated neuroprotection and improved survival of neurons in animal models [179]. Adenoviral HSP70 gene therapy has also shown protective effects in the brain. Furthermore, HSP70 protein can still protect neurons when delivered several hours after stroke onset. However, viral vector-mediated gene therapy has limitations in terms of restricted transfection extent. Delivery methods with improved efficiency are necessary for clinical translation. Drug administration to induce HSP70 expression is one potential approach, and geldanamycin has shown promise in reducing lesion size in experimental stroke models [180]. Nonviral vector systems, such as plasmid DNA complexed with cationic lipids or fusion of HSP70 with the TAT protein from HIV, are also being explored for HSP70 delivery [181]. A cancer cell membrane-derived nanocarrier (mCas9-sGNRs) efficiently delivers CRISPR/Cas9 and gold nanorods (GNRs) for synergistic photothermal/gene therapy, inducing significant antitumour effects. The downregulation of HSP70, achieved through gene editing of surviving, enhances the therapeutic efficacy of GNR-mediated PTT, showcasing the potential of this versatile nanoplatform in bimodal synergistic cancer treatment [182]. The photoactivatable RNAi system, PEI-SWNT/pHSP-shT, enables precise optogenetic control of gene knockdown in tumour cells, targeting human telomerase reverse transcriptase. Triggered by NIR light, this system offers potential advancements in cancer gene therapy, utilising HSP70B′-promoter-driven RNAi to enhance therapeutic precision [183].

A chlorin e6-encapsulated fluorinated dendrimer, HSP70-promoter-driven CRISPR/Cas9 system (F-PC/pHCP), achieves permanent genomic disruption of PD-L1, preventing immune escape and inducing immunogenic cell death. This specific promoter-driven CRISPR/Cas9 system not only exhibits potent anticancer efficacy but also reprograms the immunosuppressive TME, offering a promising strategy for gene therapy and enhanced cancer immunotherapy [184].

HSP70, Hsp90 and cochaperones are involved in stabilising and localising mutant p53 in cancer cells. These chaperone complexes prevent mutant p53 from entering the nucleus, where it exerts tumour-suppressive functions, and mask its nuclear localisation signal. Treatments targeting these complexes, like geldanamycin, show promise in promoting mutant p53 degradation. Conversely, wild-type p53 can accumulate in the cytoplasm in certain cancers due to HSP70 family interactions, resisting degradation and impairing DNA damage response [185]. Proteins like mot-2 further influence wild-type p53 localisation. Cellular stress stabilises p53, promoting its nuclear accumulation. Peptides from p53's tetramerization domain aid its nuclear localisation, offering a potential therapeutic strategy [186]. Overall, molecular chaperones, including HSP70 and Hsp90, are crucial for regulating mutant and wild-type p53 in cancer, suggesting them as potential targets for therapy.

These approaches aim to accomplish widespread distribution and expression of HSP70 in the brain. HSP70-based gene therapy holds potential for treatment of brain ischemia and related conditions [187].

#### 5.2.10. HSP70 Vaccine

Since the groundbreaking discovery by Blachere and colleagues in 1997 of HSP70–peptide complexes inducing antigen-specific CD8+ T-cell responses, HSP70 has emerged as a promising adjuvant in the cancer vaccine therapies [189, 190]. Various studies showcased the effectiveness of HSP70-based vaccines in conjunction with tumour-associated antigens (TAAs), tumour-specific antigens (TSAs) and diverse vaccine types (DNA, protein and tumour cell lysate) across different cancers. Noteworthy examples include HPV-16 E7 fused with HSP70 for cervical cancer, Dickkopf-1 (DKK1)-HSP70 DNA vaccines in multiple myeloma and AFP-HSP70 for hepatocellular carcinoma, demonstrating both prophylactic and therapeutic antitumour effects [191, 190]. Additionally, research explores innovative delivery systems such as nanoemulsion-encapsulated protein vaccines for enhanced immune responses [190, 193]. These studies collectively illustrate promising directions for HSP70-based vaccine optimisation in content, formulation and delivery methods in the realm of cancer immunotherapy. Moreover, diverse vaccination strategies employing HSP70, including conjugated vaccines, DC-based vaccines and AAV vector vaccines, exhibit significant potential for both prophylactic and therapeutic benefits across various cancer types [194].

Study by Geng and his colleagues explored combining HSP70 vaccine therapy with gene therapy targeting PD-1 to treat metastatic melanoma. While the HSP70 vaccine alone initially reduced metastasis, long-term efficacy was limited. B7-H1, a negative regulator of immune response, was identified as a key factor in tumour resistance, with IFN-g stimulation contributing to its upregulation. Blockade of B7-H1 with soluble PD-1 significantly enhanced the vaccine's efficacy, reducing metastatic foci and improving survival. This combination therapy also increased tumour-specific CTL activity and promoted a favourable TME. The hydrodynamics-based gene delivery of PD-1 showed sustained therapeutic effects with reduced side effects compared to antibodies, indicating potential for enhancing cancer immunotherapy [195].

#### 5.2.11. Ferroptosis and Pyroptosis

The combination of HSP70 with ferroptosis involves intricate molecular interactions that impact various physiological processes, including iron metabolism, lipid peroxidation and redox homeostasis. HSP70 has been shown to regulate ferroptosis through pathways like the GSH/GPX4 axis and lipid peroxidation, influencing the occurrence and development of ferroptosis in different contexts such as tumourigenesis and neurodegenerative diseases [196].

Insufficient RFA or heat stress induces high HSP70/HIF-1α expression, with HSP70 knockdown reducing HIF-1α SUMOylation and activating ferroptosis. This indicates HSP70's role in inhibiting ferroptosis, leading to lung cancer recurrence post-RFA. The findings shed light on potential therapeutic targets to suppress lung cancer recurrence and warrant further clinical investigation [197]. Additionally, the interplay between HSPs and ferroptosis-related markers like SLC7A11 and ACSL3 further underscores the regulatory role of HSPs in modulating ferroptotic pathways.

Research across various fields has delved into the interaction between HSP70 and pyroptosis, revealing the intricate mechanisms governing cell death processes. HSP70, a versatile chaperone protein, is known to modulate pyroptosis by influencing mitochondrial function, oxidative stress and inflammatory pathways. Studies have demonstrated that HSP70 can mitigate pyroptosis-related damage by suppressing mitochondrial reactive oxygen species (ROS) production and preserving microvascular barrier integrity following exposure to bacterial toxins. This highlights its protective role in mitigating pyroptosis-induced harm [198].

Furthermore, research suggests that HSP70 can alleviate sepsis-induced cardiomyopathy by attenuating mitochondrial dysfunction-initiated NLRP3 inflammasome-mediated pyroptosis in cardiomyocytes. The inhibitory effect of HSP70 on pyroptosis involves interactions with key molecules such as NLRP3, caspase-1 and IL-1β, ultimately leading to a reduction in pyroptotic cell death. These findings underscore the crucial role of HSP70 in regulating pyroptosis by modulating mitochondrial function, inflammatory responses and cell survival pathways. Further exploration of the interplay between HSP70 and pyroptosis holds promise for identifying potential therapeutic strategies for conditions characterised by dysregulated cell death processes [199].

A recent study investigated the role of TTC4 in sepsis-induced acute lung injury (ALI) focussing on its interaction with HSP70 and its impact on pyroptosis. Downregulation of TTC4 was observed in both patients with sepsis-induced ALI and a mouse model. Inhibition of TTC4 promoted inflammation and lung injury, while its overexpression reduced pyroptosis in macrophages by inhibiting mitochondrial damage. TTC4 induced HSP70 expression, which in turn attenuated NLRP3-induced pyroptosis. Moreover, the m6A-forming enzyme METTL3 reduced TTC4 stability, suggesting a regulatory mechanism in sepsis-induced ALI. These findings highlight TTC4 as a potential therapeutic target for treating sepsis-induced lung injury, particularly through its interaction with HSP70 to mitigate pyroptosis [200].

**Figure 12:**
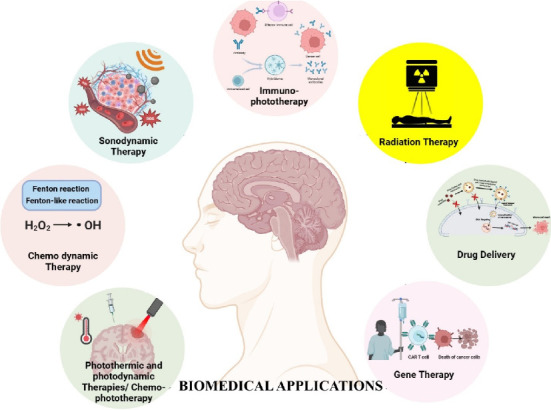
Schematic depiction of biomedical applications in GBM.

The extensive role of HSP70 in cancer has led to diverse research avenues. Studies show its impact on tumour development, resistance to therapy and potential as a predictive marker. Innovative treatments combining HSP70 modulation with therapies like phototherapy, chemophototherapy and gene therapy highlight promising directions for cancer treatment. HSP70's involvement in immunomodulation and its utilisation in targeted therapies signify its potential in revolutionising cancer treatments as portrayed in Figure 12. Understanding HSP70's multifaceted roles opens doors for advanced therapeutic strategies, holding significant promise for future cancer therapies.

### 5.3. Imaging Platforms/Diagnostic Application of GBM

Imaging platforms and diagnostic techniques are essential tools for accurately diagnosing and characterising GBM tumours [201]. Advanced imaging technologies, such as MRI, PET and CT scans, single-photon emission computerised tomography (SPECT), optical fluorescence imaging and photoacoustic imaging (PAI), enable medical professionals to visualise the size, location and specific features of GBM tumours. This information is crucial for determining the optimal treatment strategy and monitoring treatment response. In GBM, the ability to precisely locate the tumour, assess its size and identify any potential spread to surrounding brain tissues is vital for guiding surgical interventions and treatment planning. Additionally, imaging can help identify tumour recurrence and monitor treatment response over time, allowing for timely adjustments in the therapeutic approach.

The combination of HSP70 with Imaging Platforms/Diagnostic presents a synergistic approach in GBM treatment. By using imaging to identify and characterise GBM tumours, medical professionals can precisely target the affected areas with therapeutic interventions. Introducing HSP70 therapy at the tumour site can sensitise the tumour cells, making them more responsive to conventional treatments like chemotherapy and radiation. Moreover, imaging can be used to monitor the response to HSP70 therapy and detect any potential recurrence of the tumour. This real-time feedback allows for personalised treatment adjustments and optimisations, leading to improved patient outcomes.

#### 5.3.1. PAI

PAI and HSP70 can be used synergistically for the treatment of GBM. PAI provides imaging guidance for targeted HSP70 delivery, monitoring HSP70 expression and assessing treatment efficacy. By combining PAI with HSP70-based therapies, the accumulation of HSP70 in the tumour site can be enhanced, improving therapeutic effects. Additionally, PAI can visualise and quantify changes in HSP70 expression levels, providing real-time feedback on the treatment response. Furthermore, PAI can assess the efficacy of HSP70-based therapies by monitoring changes in tumour vasculature, oxygenation and functional parameters. This integrated approach has the potential to enhance the precision and improving effectiveness of GBM treatment strategies [202].

The research presents a ‘four-in-one' theranostic system for BCa treatment, merging PAI-guided chemo-gene-thermotherapy. This approach combines PEG-bridged polyethylenimine (PEI) with a memHSP70 receptor-targeting peptide (TKD), PPT, onto DOX-loaded oxidised mesoporous carbon nanospheres (OMCN). This system efficiently delivers therapeutic genes into cancer cells, leveraging OMCN's properties (high photothermal conversion, strong PA contrast and controlled drug loading) and the hydrophilic polymer's benefits (gene vector, pore cap control and targeting). It offers PAI, NIR/pH responsive drug/gene release and effective synergistic targeting therapy, showing superiority over single or dual therapies, promising advanced cancer treatments [203].

Zhong and his colleagues performed a study that demonstrates the use of pH-responsive Ag_2_S nanodots (Ag_2_S NDs) loaded with an HSP70 inhibitor (QE-PEG-Ag_2_S) for enhanced photothermal cancer therapy. This nanomaterial exhibits water solubility and biocompatibility, entering cells efficiently and accumulating in lysosomes. The slightly acidic tumour cell environment and lysosomal acidity, combined with PTT using NIR light (808 nm), facilitate the release of the inhibitor to counteract heat resistance in cancer cells. QE-PEG-Ag_2_S also possesses PAI capabilities, allowing precise tracking of nanomaterial accumulation in tumours and determining optimal timing for light therapy. When exposed to NIR light for 10 min, QE-PEG-Ag_2_S achieved complete tumour ablation without recurrence, highlighting its potential for effective theranostic applications in cancer treatment [117].

#### 5.3.2. SPECT

A study by Astrid and colleagues study aimed to evaluate the cytoprotective effects of recombinant human heat-shock protein 70 (rhHSP70) against oxidative stress in retinal pigment epithelial cells (ARPE-19). Pretreatment with rhHSP70 resulted in decreased IL-6 secretion, increased cell viability and reduced cell lysis following exposure to hydrogen peroxide. Cellular uptake studies revealed endocytosis of rhHSP70 by ARPE-19 cells, localising it in late endosomes and lysosomes. Intravitreal injection into porcine eyes demonstrated a substantial presence (20%) of rhHSP70 in the retinal pigment epithelium. These findings highlight the potential therapeutic application of rhHSP70 in protecting against oxidative stress, and the use of SPECT enhances the study's significance for developing targeted strategies in age-related macular degeneration (AMD) [204].

GRP78, part of the HSP70 family, is abundantly found on cancer cell surfaces, making it an attractive target for therapy and imaging. Using targeted peptides or antibodies coupled with imaging tracers like SPECT enhances tumour detection. csGRP78-targeted agents, including liposomes and polymeric micelles, display increased uptake in tumours. PET probes targeting csGRP78 enable precise imaging, particularly in different BCa subtypes. csGRP78 also finds utility in nucleic acid delivery for hindering tumour growth and in PDT, demonstrating superior efficacy in cancer cells. These applications highlight the potential of csGRP78 beyond imaging and therapy, showing promise for advanced cancer diagnostics by combining csGRP78 ligands with various imaging modalities like SPECT and CT imaging using GNRs and biosensors [205].

#### 5.3.3. CT

CT scans play a significant role in both the diagnosis and the treatment of glioblastoma. These imaging tests provide detailed visualisations of the brain, allowing healthcare professionals to identify and characterise the tumour. CT scans are used to assess the location, size and extent of the GBM, which helps in planning surgical interventions and radiation therapy. During hyperthermia treatment, HSP70 is upregulated. It plays a vital role in linking the innate and adaptive immune responses against the tumour. HSP70 helps activate immune cells, such as dendritic cells, T cells and NK cells facilitating an antitumour immune response. By combining CT scans with the assessment of HSP70 expression, healthcare professionals can make more informed treatment decisions for GBM patients. The correlation between CT findings and HSP70 levels helps evaluate treatment effectiveness and guide further therapeutic interventions. This integrated approach holds promise for personalised and targeted management of GBM, enhancing patient outcomes [206].

Effectiveness of anti-HSP70 antibody-coated gold nanoparticles (cmHSP70.1-AuNPs) in tumour-specific imaging using CT was explored by researchers. By targeting membrane-associated HSP70, these nanoparticles were observed to accumulate specifically within tumour cells, enhancing CT-based tumour visualisation. In preclinical models, cmHSP70.1-AuNPs displayed selective accumulation in malignant cells, demonstrating their potential for precise CT imaging and improving diagnostic accuracy for cancer [107].

A similar research also examined the potential of using gold nanoparticles conjugated to cmHSP70.1 antibody as a novel strategy for cancer imaging and treatment, focussing on HSP70-positive tumour cells. Complementing CT imaging techniques, the study explores the precise targeting and uptake of these nanoparticles within tumour cells. The findings reveal that cmHSP70.1-conjugated gold nanoparticles exhibit superior uptake compared to other nanoparticle types in various tumour entities. The study emphasises the importance of targeting membrane bound HSP70, which is exclusively presented in malignant cells, offering promising prospects for precise tumour imaging [106].

#### 5.3.4. MRI

MNPs were employed to precisely deliver HSP70 to glioblastoma tumours and was confirmed by MRI. MRI sequences with a magnetic field of 1.5 T or higher are used for diagnosing brain tumours and monitoring treatment in clinics. Commonly employed sequences include T1-weighted MRI (T1WI) and T2-weighted MRI (T2WI). T1WI provides detailed anatomical information, while T2WI helps detect lesions and determine tumour types. Contrast agents, such as gadolinium (Gd)-based T1 contrast agents, are often used to improve resolution and sensitivity. These agents assist in visualising the boundary information of brain tumours more clearly [207]. The biodistribution of HSP70-MNPs in a rat model of cerebral glioma was studied utilising MR imaging and magnetometry. HSP70-MNPs accumulated in the glioma, as revealed by MR scans, according to the findings. Further magnetometry showed the glioma's preferential accumulation of HSP70-MNPs over surrounding normal brain tissues. The study indicated that employing MRI-guided targeting, HSP70-MNPs may be efficiently administered to glioblastoma tumours. This approach holds promise for improving the specificity and effectiveness of immunotherapy in neuro-oncology, as it allows for precise delivery of chemotherapeutic agents directly to tumour site [135].

MRI was used to visualise tumours after SPIONs, in combination with antibodies against HSP70, exhibited effective accumulation in glioblastoma cells [208]. Nanoparticles containing metals such as gold (Au), silver (Ag) and gadolinium (Gd) nanoparticles have also shown potential in combating glioblastoma. For example, silver nanoparticles have exhibited preferential radiosensitising properties compared to gold nanoparticles. Studies have shown that silver nanoparticles can improve the effectiveness of radiotherapy in glioma cells, resulting in a significant reduction in tumour growth [209].

#### 5.3.5. PET

A novel PET tracer, TPP-PEG24-DFO[89Zr], targeting membrane HSP70 (mHSP70) exclusively exposed in various tumour types, was developed for in vivo imaging. This tracer exhibited specific binding to mHSP70-positive tumour cells, displaying high tumour-to-background contrast and renal clearance. In testing, it accurately accumulated in tumours with different mHSP70 densities, reflecting their expression profiles. Importantly, it showed no accumulation in mHSP70-negative cells. This chemically optimised tracer, TPP-PEG24-DFO[89Zr], holds promise for highly specific imaging of diverse tumour types exhibiting surface expression of HSP70, showcasing its potential for cancer PET imaging [210].

Triggering of the heat shock factor 1 (HSF-1) transcription factor is a crucial cellular response to proteotoxic cell stresses. HSF-1 regulates the expression of HSPs, including HSP70, which play a vital role in cellular protection and stress adaptation [211]. An innovative [124I]iodide-pQHNIG70 PET-reporter system utilises an inducible HSP70 promoter to monitor HSF1/HSP70 activation in response to drugs like 17-AAG. PET imaging visualises and quantifies these molecular processes, offering insights into drug-induced stress responses. This noninvasive technique tracks real-time HSP70 expression, aiding in treatment assessment and understanding cellular reactions to drugs, particularly in diseases like cancer. This HSP70-PET combination provides a sensitive means to study drug-induced cellular stress and refine personalised therapies [212].

Research conducted by Anil and his colleagues explored the correlation between thermal treatments and HSP70 expression in prostate tumours. The study found a correlation between cell viability and HSP70 levels using modified metastatic prostate tumour cells and heating temperatures. This suggests HSP70's potential as a marker for planning thermal therapy in prostate cancer. The study envisions a PET-MR imaging approach for treatment planning, real-time monitoring and post-therapy assessment, aiming to evaluate tumour response at a cellular level [213].

#### 5.3.6. Optical Fluorescence Imaging

NK cell therapy shows promise for treating GBM. However, the limited supply of fully functional cells constrains the promise of this developing technology. The efficiency of NK cells that had been developed and treated with IL-2 and HSP70 both in vitro and in vivo was examined in present research. NK cells were administered intracranially as well as systemically to both treated and untreated NK cells to examine the potential anticancer activity in the in vivo rat GBM models after the function of NK cells had been evaluated in vitro using proliferation and cytotoxicity tests. Using fluorescent imaging, the biodistribution of NK cells was assessed, demonstrating their penetration into the tumour. Injections of cytokine-treated NK cells intravenously and systemically significantly decreased tumour cell growth and increased survival rates in animal GBM models. The antitumour actions of NK cells were validated by histopathological investigation [214].

Recent researchers used green fluorescent protein (GFP) to monitor HSP70 expression in cultured endothelial cells. Cells were exposed to controlled heat (42°C) and then shifted to normal temperature (37°C). Results showed two peaks in HSP70 expression at around 3- and 12-h post-heat shock, with HSP70 gradually moving from the cell cytoplasm to the nucleus between 6 and 16 h. This research effectively combined molecular cloning and imaging, providing insights into HSP70 kinetics and its intracellular translocation [215].

In a study assessing laser surgical ablation's effects, researchers employed optical imaging to gauge the HSP70 expression as an indicator of tissue damage beyond the target area. Using a transgenic mouse model with HSP70-driven luciferase and GFP, various laser wavelengths were tested. The findings revealed that at 6.10 μm wavelength, the tissue sustained less damage and exhibited lower epidermal hyperplasia than at 2.94 μm and 6.45 μm. This highlights the potential superiority of the 6.10-μm wavelength in laser ablation for precise cancer therapy applications, showing promise for minimising collateral damage in cancer treatment procedures [216].

## 6. Clinical Trials and Patents

Clinical trials on HSP70 explore its potential in cancer treatment along with immunotherapy. Clinical trials portrayed in Table 2 assess HSP70-based approaches like vaccines, inhibitors and antibodies across various cancers, investigating its impact on immune responses, tumour regression and diagnostic potential for circulating tumour cells (CTCs). Research also examines HSP70's role in radiation-induced fibrosis and genetic influences on cancer susceptibility. While promising, more research is needed to understand HSP70's full clinical impact across different cancers and treatments.

### 6.1. Translational Timelines and Challenges

While some trials, such as those involving HSP70 DNA vaccines, have demonstrated lesion regression and weak immune responses, heterologous prime-boost regimens offer a promising strategy for enhanced efficacy. Similarly, the diagnostic potential of HSP70 in isolating CTCs could revolutionise cancer detection and monitoring by enabling real-time evaluation of tumour dynamics. However, translating these findings into clinical practice requires substantial validation across larger cohorts and diverse patient populations.

The timeline for implementing HSP70-based interventions into routine practice remains uncertain due to several bottlenecks:

  Limited Robustness in Early-Stage Results: Many HSP70-related trials have produced promising preclinical or early-phase outcomes but lack scalability for larger, multicentre trials [59].
  Complex Immune Dynamics: The dual role of HSP70 in both tumour promotion [58] and suppression [32] necessitates precise patient stratification to optimise therapeutic outcomes.
  Cost and Manufacturing Concerns: Developing cost-effective production methods for HSP70-based therapeutics and diagnostics is critical for scalability [217].

### 6.2. Regulatory Challenges

HSP70-based interventions, particularly those involving nanocarriers or conjugated systems, face additional regulatory hurdles inherent to nanomedicine. Challenges in advancing HSP70-based nanomedicines include the need for detailed physicochemical profiling, such as stability and in vivo behaviour, to meet regulatory standards. Defining dynamic surface modifications, like PEGylation, is particularly critical for ensuring consistency and efficacy. Additionally, the long-term toxicity and potential off-target effects of these nanocarrier systems remain unclear, posing significant hurdles for approval. The absence of harmonised global regulations further complicates the process, requiring developers to navigate varying guidelines from bodies like the FDA, EMA and ICMR [218]. A multidisciplinary approach, collaboration with regulators, and computational modelling can streamline approvals and accelerate transitions from preclinical to clinical stages.

The patents related to HSP70 encompass innovative methodologies, treatments and diagnostic tools targeting this protein. The patents enlisted in Table 3 elucidate novel approaches such as screening for anticancer agents, therapeutic interventions, inhibitors, antibodies, diagnostic assays and fusion proteins involving HSP70. They highlight the significance of HSP70 in cancer therapy, immunotherapy, diagnostics and its potential role in modulating immune responses and treating various diseases.

## 7. Industrial Scale-Up

In the process of industrial scale-up for HSP70-conjugated nanoparticles, researchers navigate through a series of challenges and optimisation steps to translate promising research into practical therapies. Initially, laboratory synthesis involves fine-tuning parameters such as nanoparticle size, surface functionalisation and HSP70 attachment efficiency on a small scale. However, during industrial scale-up, challenges arise due to increased batch sizes, necessitating adaptation of the synthesis process to accommodate larger quantities of nanoparticles. This requires the use of larger reactors, purification systems and handling equipment to meet the goal of producing kilograms or even tons of nanoparticles per batch [128].

Quality control becomes critical during industrial scale-up, with robust protocols in place to monitor particle size distribution, surface charge and HSP70 loading. Ensuring consistent properties across batches is critical for clinical applications. Moreover, cost-efficiency becomes a primary concern, prompting researchers to explore cost-effective raw materials, efficient processes and recycling/reuse strategies to optimise production economics [107, 135].

One innovative approach for HSP70 delivery involves cell-penetrating peptides (CPPs), such as PepFect14 [231], which efficiently transfect HSP70 into cells, offering therapeutic potential for diseases like Parkinson's and Alzheimer's. Another platform, Fv-HSP70, targets and delivers Hsp72 directly into damaged cells to prevent apoptosis. FA is an effective and feasible therapy for lung cancer, but accelerated progression of residual non-small cell lung cancer (NSCLC) after incomplete RFA has frequently been reported.

A previous study reported that HSP70 and HIF-1α were highly expressed in areas with incomplete RFA. Therefore, we sought to elucidate the regulatory effect of the HIF-1α/HSP70 pathway on lung cancer recurrence after incomplete RFA. In this study, we found that knockdown of HSP70 can reduce sumo 1, sumo 2/3 (marker of SUMOylation) of HIF-1α and inhibit A549 cell proliferation and migration under heat stress conditions (used to simulate incomplete RFA in vitro). We observed that knockdown of HSP70 altered the expression of ferroptosis-related proteins and genes (SLC7A11 and ACSL3), and the RNA-seq results showed that knockdown of HSP70 activated the ferroptosis pathway, further confirming that HSP70 regulates ferroptosis. In summary, HSP70, via HIF-1α SUMOylation, inhibited ferroptosis, inducing lung cancer recurrence after RFA. The study reveals a new direction for further research on therapeutic targets to suppress lung cancer recurrence and provides a theoretical foundation for further clinical studies [197]. Additionally, intranasal administration of exogenous HSP70 shows promise for neuroprotection in conditions like Alzheimer's. From an industrial perspective, both intravenous and intranasal administration routes offer scalable delivery technologies, with potential applications tailored to specific therapeutic needs and target tissues. One study has demonstrated the use of exogenous HSP70 conjugated to the HIV TAT protein to improve brain delivery following intravenous administration [232]. HSP70 has been shown to protect against dopaminergic denervation and modulate neuroinflammatory response in a 6-OHDA rat model. This route of administration bypasses the BBB and can potentially deliver HSP70 directly to the central nervous system [233].

Scalable synthesis routes, such as the solgel method, microwave-assisted synthesis and continuous flow reactors, offer avenues for efficient nanoparticle production at an industrial scale. Automated purification systems and sterilisation procedures handle large volumes, ensuring safety and compliance with regulatory standards for medical use. Long-term stability optimisation, regulatory compliance and collaboration with industry partners further contribute to the successful commercialisation of HSP70-conjugated nanoparticles. Through concerted efforts to overcome technical, economic and regulatory challenges, industrial scale-up endeavours pave the way for the widespread adoption of these innovative nano-enabled therapies in clinical settings [234].

## 8. Future Directions

The future of HSP70-targeted therapies lies in their ability to bridge the gap between experimental innovation and clinical application. Advancing these approaches requires addressing key challenges, refining therapeutic designs and emphasising their translational potential to revolutionise cancer care [235].

One of the foremost priorities is optimising delivery systems to overcome biological barriers such as the BBB, particularly in treating glioblastoma [236]. The development of multifunctional nanocarriers that leverage HSP70's tumour-specific overexpression while incorporating active transport mechanisms, such as receptor-mediated transcytosis, holds significant promise for enhancing therapeutic efficacy [237].

Patient stratification will play a pivotal role in ensuring treatment success. Personalised medicine approaches that utilise biomarkers to identify tumours with high HSP70 expression can maximise therapeutic outcomes. This precision-based strategy will enable clinicians to tailor treatments to individual tumour profiles, improving efficacy and minimising off-target effects [238].

Resistance mechanisms represent another critical area of focus. Combining HSP70-targeted therapies with other modalities, such as immune checkpoint inhibitors or metabolic pathway modulators, offers a synergistic approach to overcoming adaptive tumour survival strategies. These combinations could further enhance therapeutic responses and delay or prevent resistance [24].

Changes in eHSP70 expression offer additional opportunities for clinical translation. eHSP70 expression may differentiate cancer types and provide real-time insights into therapeutic efficacy and potential recurrence. Integrating artificial intelligence (AI) tools, such as quantitative proteomics and machine learning, can construct predictive cancer networks based on eHSP70 expression, advancing precision oncology [58].

To ensure clinical translation, scalability and regulatory considerations must be prioritised. Establishing standardised manufacturing protocols and engaging regulatory bodies early in the development process will streamline the approval and deployment of HSP70-targeted nanotechnologies [17].

Finally, integrating imaging and therapeutic capabilities into single nanoplatforms can enhance early diagnosis and real-time monitoring, making these theranostic systems invaluable in precision oncology. These advancements hold the potential to redefine cancer treatment, offering a future of more effective, personalised and less toxic therapies [238].

## 9. Conclusion

HSP70-targeted nanotechnologies are at the forefront of advancements in cancer therapy, offering unparalleled specificity, efficient drug delivery and improved diagnostic accuracy. By leveraging HSP70's tumour-specific overexpression, these approaches effectively integrate imaging and therapeutic modalities, enabling precise targeting while minimising off-target effects. The incorporation of HSP70-targeted nanocarriers into cancer treatment paradigms has the potential to significantly enhance therapeutic efficacy and patient outcomes.

These technologies showcase the ability to seamlessly combine diagnostic and therapeutic functions, opening new avenues for precision oncology. The integration of nanocarrier systems with HSP70 targeting not only addresses the need for effective treatment options but also highlights their adaptability for various cancer types and therapeutic strategies. From advancing imaging techniques to enhancing drug delivery, these innovations are transforming cancer care into a more personalised and effective practice.

With continued development and refinement, HSP70-targeted nanotechnologies hold immense potential to redefine oncology, offering hope for better survival rates and improved quality of life for patients. These breakthroughs underscore the transformative role of HSP70 in driving innovation in cancer treatment and shaping the future of personalised medicine.

## Figures and Tables

**Table 1:** Nanoparticle HSP70 synergies in cancer therapy: formulations, cancer types and therapeutic insights.

Nanoparticles		Formulations	Type of cancer	HSP70 role inference	Advantage of HSP70	Comparison with traditional therapy	Reference
SPION		SPION-conjugated cmHSP70.1 monoclonal antibodies	Glioblastoma	SPION nanoparticles, conjugated with cmHSP70.1 antibody, demonstrated specific binding to HSP70 in GBM. In vitro and in vivo studies confirmed targeted delivery, enhancing MRI contrast. Irradiation increased HSP70 expression, improving nanoparticle uptake	SPION-cmHSP70.1 conjugates, guided by HSP70, offer enhanced GBM-specific MRI contrast, showcasing potential for advanced diagnostics and therapy	Without HSP70 targeting, SPION formulations lose cancer-specific targeting, leading to off-target effects and reduced nanoparticle uptake, compromising MRI contrast and therapeutic efficacy, especially in glioblastoma. In GrB-SPIONs for melanoma, the lack of HSP70 diminishes targeting, reducing therapeutic delivery and weakening synergy with immune checkpoint inhibitors, impairing apoptosis induction and tumour control. For SPIONMTO, absence of HSP70 limits immunogenic response, reducing immune activation, local drug delivery and increasing systemic toxicity. Without HSP70, SPIONs fail to efficiently deliver antigens and activate T cells, reducing antitumour immunity and survival benefits. Additionally, bPEI-SPIONs without HSP70 are less effective at antigen delivery to dendritic cells, limiting immune responses and potential in cancer immunotherapy	[101]
		GrB-SPIONs	Melanoma	GrB-SPION nanocomplexes, targeting HSP70 in melanoma cells, exhibited specific therapeutic efficacy. Combining them with immune checkpoint inhibitors enhanced anti-tumour effects, suggesting clinical potential	Targeting melanoma with HSP70-specific SPIONs loaded with granzyme B induces apoptosis, delaying tumour progression. Combining this nanocomplex with immune checkpoint inhibitors synergistically enhances therapeutic efficacy		[102]
		SPION^MTO^	All types of cancer	SPION^MTO^ induces immunogenic cell death in HT-29 cells, releasing HSP70. Magnetic targeting ensures localised drug delivery, minimising systemic toxicity	HSP70's role in immunogenic response suggests its potential to enhance therapeutic efficacy against cancer		[103]
		HSP70-SPIONs	Glioblastoma	HSP70-SPION nanosuspensions offer potent MR negative contrast. They efficiently deliver antigens, boosting T-cell activity. In vivo, they enhance overall survival, promoting antitumour immunity	Magnetic nanoparticles carrying HSP70 show promise for enhanced cancer treatment		[104]
		bPEI-SPIONs	All types of cancer	SPIONs conjugated with HSP70 protein improve cancer immunotherapy. These nanoparticles showed efficient antigen delivery to dendritic cells (DCs) and enhanced immune responses in in vitro and in vivo experiments	Potential therapeutic use of HSP70-conjugated nanoparticles for boosting antitumour immunity		[105]

Metal nanoparticles	Gold nanoparticles	cmHSP70.1 antibody-conjugated gold nanoparticles	All types of cancer	Highly malignant HSP70 membrane-positive cancer cells specifically take up the cmHSP70.1-conjugated gold nanoparticles, whereas control and empty nanoparticles only show limited uptake	Study confirms HSP70 specificity by demonstrating uptake in HSP70-expressing cells and negligible uptake in HSP70 knockout cells	Without HSP70 targeting, the formulations lose key therapeutic benefits. cmHSP70.1-AuNPs would lack specificity for HSP70-positive cancer cells, reducing uptake and targeting potential for imaging and therapy. Fe3O4-Au nanoparticles (TPP-PEG4-FeAuNPs) would fail to efficiently target TNBC cells, impairing radiation sensitisation and ROS-induced apoptosis. PtFe NP's photothermal therapy and bioreduction efficiency would decrease without HSP70. DIO-NP would lose its protective role in pancreatic cancer cells, affecting both biocompatibility and therapeutic efficacy. PEI-Mn_0.5_Zn_0.5_Fe_2_O_4_ nanoparticles would be less effective in inhibiting liver cancer and suppressing xenograft growth, as HSP70 enhances responses to hyperthermia and radiation. ZIPP-Apt:DOX/siHSPs would show diminished tumour-specific accumulation and therapy efficacy without HSP70. MP-AgNPs would fail to induce apoptotic cell death, losing anticancer potency, while AgNPs would lack the protective role of HSP70 in TNF-α-induced damage. AgNPs-G would lose the ability to trigger calreticulin exposure and HSP70 release, reducing cytotoxicity in breast cancer. AgAu NPs would not effectively promote apoptosis or HCT116 cell death without HSP70. Ag_2_S NDs would show reduced photothermal therapy efficacy, hindering tumour ablation. Lastly, CuONPs would experience increased toxicity due to a lack of HSP70's protective role. In essence, HSP70 is crucial for the targeting, therapeutic efficacy and minimising off-target toxicity of these formulations. Without HSP70, these nanotube formulations would lose key therapeutic advantages. AgNPs/TNTs would become less effective in detecting HSP70 for cancer diagnostics. The siRNA-GOx/GNR@HA gold nanorod system would lose its ability to enhance low-temperature photothermal therapy, increasing toxicity. Ox-MWCNTs would be less effective in breast cancer therapy, as the lack of HSP70 overexpression would hinder tumour eradication and immune response. Bi2S3–Au nanorods would induce less apoptosis, weakening photothermal treatment. AuNRs would fail to induce apoptosis effectively during photothermal therapy, and MWNTs would see reduced efficacy in cancer treatment due to impaired HSP expression reduction. The absence of HSP70 reduces targeting precision, therapeutic potency and overall success	[106]
		cmHSP70.1-AuNPs	All types of cancer	cmHSP70.1 antibody-conjugated gold nanoparticles exhibit selective uptake in HSP70-positive cancer cells, suggesting potential for targeted cancer spectral CT imaging	Promising role for cmHSP70.1-AuNPs in precise tumour targeting, with potential applications in both imaging and therapeutics		[107]
		FeAuNPs	Triple-negative breast cancer	Hybrid Fe3O4-Au nanoparticles (TPP-PEG4-FeAuNPs), targeting HSP70, improve TNBC cell targeting and sensitise to radiation, triggering apoptosis via ROS induction, showcasing potential for enhanced cancer therapy	Nanoparticles, serving as MRI contrast agents, accumulate in HSP70-positive tumour cells, inducing cell cycle arrest and DNA damage		[108]
	Iron nanoparticles	PtFe NP	All types of cancer	Nanostructure CV@PtFe/(La-PCM) combines NIR-triggered photothermal therapy and HSP70-mediated bioreduction, amplifying endogenous H_2_O_2_ for efficient cancer treatment	HSP70's tumour-specific overexpression aids precise nanoparticle delivery, enhancing efficacy in cancer nanocatalytic therapy		[109]
		DIO-NP	Pancreatic cancer	HSP70 exhibits a dynamic response in pancreatic cancer cells exposed to DIO-NPs, playing a pivotal role in cellular protection, while DIO-NPs demonstrate dual potential biocompatibility for MRI contrast at lower doses and cytotoxicity at higher doses, suggesting their application in cancer theranostics	HSP70, with its dynamic regulation, acts as a cellular protector in pancreatic cancer cells		[110]
		PEI-Mn0.5Zn0.5Fe2O4 nanoparticles	All types of cancer	PEI-Mn_0.5_Zn_0.5_Fe_2_O_4_ nanoparticles, incorporating HSP70 gene, exhibit effective in vitro liver cancer cell inhibition and in vivo xenograft growth suppression through heat/radiation induction	HSP70 enhances therapeutic effects by responding to hyperthermia and radiation		[111]
		ZIPP-Apt:DOX/siHSPs	All types of cancer	ZIPP-Apt:DOX/siHSPs, a targeted theranostic nanocomplex with zinc-doped iron oxide core, AS1411 aptamer and HSP70/HSP90 siRNAs, enhances tumour-specific accumulation, magnetic hyperthermia, and chemotherapy-induced cell death	HSP70 enhance cellular responses to therapy, facilitate conformational changes in signalling pathways, suppress the release of proinflammatory factors and play an antiapoptotic role		[112]
	Silver nanoparticles	MP-AgNPs	All types of cancer	Macrolepiota procera-derived silver nanoparticles (MP-AgNPs) synthesised through green chemistry demonstrate potent anticancer effects, particularly inhibiting HSP70 in cancer cell lines	The nanoparticles induce apoptotic cell death, showcasing their potential as effective HSP70 inhibitors for cancer treatment		[113]
		Ag-nps	All types of cancer	Silver nanoparticles (AgNPs) at 1 μg/mL protect against TNF-α-induced cell damage, inducing HSP70 expression like heat shock treatment	Advantage of HSP70 induction by silver nanoparticles lies in its protective role against TNF-α-induced cell damage		[114]
		AgNPs-G	Breast cancer	AgNPs-G show dose-dependent cytotoxicity against breast cancer cells, inducing cell death and interfering with the cell cycle. AgNPs-G treatment triggers calreticulin exposure and HSP70 release	In vivo, AgNPs-G vaccination reduces tumour weight and enhances mouse survival, highlighting potential therapeutic impact on cancer through HSP70		[115]
		AgAu NPs	All types of cancer	AgAu NPs induce dose-dependent cytotoxicity, triggering mixed cell death in P53-deficient cells, while promoting HCT116 apoptotic death with increased extracellular HSP70	Study suggests versatile effects of AgAu NPs on programmed cell death and highlights potential therapeutic implications for cancer through HSP70		[116]
		Ag2S NDs	All types of cancer	pH-responsive Ag2S nanodots loaded with HSP70 inhibitor (QE-PEG-Ag2S) enhance photothermal cancer therapy by reducing heat resistance in tumour cells	Nanoagent achieves complete tumour ablation with NIR light irradiation, offering a promising strategy for HSP70-targeted theranostic applications in cancer treatment		[117]
	Copper nanoparticles	CuONPs	All types of cancer	CuONPs exposure in fish induces dose-dependent toxicity, elevating biochemical markers and upregulating proinflammatory, heat shock, apoptosis and oxidative stress genes	HSP70's induction reflects its role in mitigating the toxic effects of CuONPs, highlighting its advantageous function in cellular homeostasis and stress adaptation		[118]
Nanotubes	AgNPs/TNTs		All types of cancer	Sensitive biosensor for tumour marker HSP70 using titanium dioxide nanotubes and silver nanoparticles, hinting at potential cancer diagnostic and therapeutic applications of nanoparticles	AgNPs contribute to the overall sensitivity and efficacy of HSP70 detection, showcasing the versatility of nanoparticles in biosensing applications and potentially extending their utility to cancer therapeutics		[119]
Gold nanorods	siRNA-GOx/GNR@HA		All types of cancer	Multifunctional gold nanorod-based nanosystem, targeting B7-H3 and depleting HSP70, enables low-temperature photothermal therapy for cancer with high treatment effectiveness and minimal systemic toxicity	Targeting HSP70 in the gold nanorod-based nanosystem enhances low-temperature photothermal therapy, promoting efficient cancer ablation with minimal systemic toxicity		[120]
Carbon nanotubes	Ox-MWCNTs		Breast cancer	Oxidised multiwalled-carbon nanotubes (ox-MWCNTs) with hyperthermia treatment show promise for breast cancer therapy, resulting in complete tumour eradication, increased median survival and upregulated HSP70 expression, suggesting a potential future in breast cancer treatment	HSP70 overexpression enhance the therapeutic effect by promoting cell survival, immune response modulation, and cellular stress adaptation, contributing to the efficacy of the treatment in breast cancer		[121]
Nanorods	Bi_2_S_3_–Au nanorods		All types of cancer	Bismuth sulphide (Bi_2_S_3_) nanorods, particularly Bi_2_S_3_–Au heterojunctions, exploit deep-level defects for potent photothermal cancer therapy, inducing elevated HSP70 expression and increased apoptosis for effective treatment guided by computed tomography imaging	Upregulation of HSP70 by Bi_2_S_3_–Au nanorods enhances photothermal cancer therapy, potentially amplifying cellular stress response for more effective tumour inhibition		[122]
Gold nanorods	AuNRs		All types of cancer	Gold nanorods (AuNRs) induce cancer cell apoptosis via PPTT. Lower HSP70 levels in Huh7.5 cells lead to increased apoptosis, suggesting a potential strategy for enhancing cancer treatment efficacy	The lower levels of HSP70 in Huh7.5 cells provide an advantage in inducing increased apoptosis during plasmonic photothermal therapy		[123]
Carbon nanotubes	MWNTs		All types of cancer	Multiwalled carbon nanotubes (MWNTs) with laser irradiation reduce heat shock protein (HSP) expression, enhancing cancer cell treatment	Reducing HSP expression with MWNTs and laser irradiation enhances cancer treatment, potentially improving therapeutic efficacy for better outcomes		[124]

Liposomes	NL-HSP70.PC-Fc		Cancer vaccines	Encapsulating dendritic cell-tumour fusion cell-derived HSP70.PC-Fc in nanoliposomes (NL-HSP70.PC-Fc) enhances bioavailability and tumour immunogenicity, demonstrating improved stability and increased antitumour immune responses in vivo	NL-HSP70.PC-Fc shows promise for advanced HSP-based tumour vaccines, warranting further investigation for potential clinical applications	Without HSP70, these liposome formulations would lose key therapeutic benefits. NL-HSP70.PC-Fc would have reduced tumour immunogenicity and bioavailability, weakening its role in cancer vaccines. Magnetic cationic liposomes (MCL) would fail to enhance tumour immunity, diminishing their effectiveness for bone metastatic prostate cancer. Doxorubicin liposomes would lose the ability to reduce cancer cell resistance to heat, limiting their effectiveness in combination therapies and apoptosis induction. Overall, the absence of HSP70 compromises delivery, immune activation and treatment outcomes	[125]
	Magnetic cationic liposomes		Bone metastatic prostate cancer	Magnetic cationic liposomes (MCL) with hyperthermia induce significant tumour suppression and enhanced tumour immunity, particularly through increased HSP70 expression, making them a promising approach for treating bone metastatic prostate cancer	The upregulation of HSP70 in response to magnetic cationic liposome (MCL) therapy offers an advantage by enhancing tumour immunity, potentially improving the therapeutic efficacy		[126]
	Doxorubicin liposomes		All types of cancer	Combination of radiofrequency ablation with adjuvant liposomal quercetin reduces HSP70 expression, enhancing coagulation size and promoting apoptosis in a rat tumour model, indicating potential improvements in cancer treatment	By downregulating HSP70, the resistance of cancer cells to heat is reduced, leading to improved therapeutic outcomes in cancer treatment		[127]

Quantum dots	Cadmium selenide quantum dots (QDs)		All types of cancer	CdSe nanoparticles, coupled with p53 and HSP70 antibodies, exhibit targeted interaction with glioblastoma cells, showcasing potential for cancer imaging and monitoring apoptotic responses	Promising strategy for cancer cell identification and monitoring, emphasising pro-apoptotic proteins like p53 and HSP70, suggesting applications in cancer imaging and detection	Without HSP70, these quantum dot formulations lose critical functions. CdSe quantum dots would fail to target glioblastoma cells, reducing imaging and apoptotic monitoring potential. Silicon-based quantum dots would lack the protective HSP70 response to oxidative stress, impairing recovery. PAGD electrochemical immunosensors would lose sensitivity in detecting depression markers, limiting diagnostic accuracy. In all cases, the absence of HSP70 reduces both therapeutic and diagnostic effectiveness	[128]
	Silicon-based quantum dots		All types of cancer	Silicon-based quantum dots induced oxidative stress in renal tissue, causing structural damage. The study highlighted a time-dependent increase in HSP70 expression, emphasising the role of heat shock proteins in responding to cellular stress	Involvement of heat shock proteins in the cellular stress response and potential tissue recovery mechanisms		[129]
	Polyaniline functionalized graphene quantum dots (PAGD)		All types of cancer	PAGD-based electrochemical immunosensor detects depression marker HSP70 with high sensitivity (0.05 ng/mL) and wide linearity (0.0976–100 ng/mL), offering a convenient diagnostic tool for early clinical screening	HSP70-based electrochemical immunosensor provides a sensitive and reliable method for detecting depression markers		[130]

Selenium nanoparticles	Se@PEI@siRNA		All types of cancer	Se@PEI@siRNA nanoparticles selectively induce apoptosis in cancerous HepG2 cells by silencing HSP70, demonstrating low cytotoxicity and effective cellular uptake. This highlights their potential for targeted cancer therapy using selenium-based nanoparticles and siRNA	By silencing the HSP70 gene using selenium nanoparticles combined with polyethylenimine and siRNA, the study induces cancer cell apoptosis through caspase-mediated pathways, increased ROS levels, and modulation of ROS-mediated signalling pathways	Without HSP70, Se@PEI@siRNA nanoparticles would lose their ability to selectively induce apoptosis in cancer cells. HSP70 silencing is essential for activating caspase pathways and ROS-mediated signalling, which are critical for the therapeutic efficacy of these nanoparticles. This would reduce their cancer-targeting ability and increase off-target effects	[56]

Dendrimer	ZIPP-Apt:DOX/siHSPs		All types of cancer	ZIPP-Apt:DOX/siHSPs, a dendrimer-based nanocomplex, efficiently targeted HSP70 in cancer therapy, combining magnetic hyperthermia and chemotherapy for enhanced cell death	HSP70 depletion by ZIPP-Apt:DOX/siHSPs nanocomplex sensitises cancer cells to combined magnetic hyperthermia and chemotherapy, enhancing therapeutic efficacy	Without HSP70, ZIPP-Apt:DOX/siHSPs would lose its ability to sensitise cancer cells to combined magnetic hyperthermia and chemotherapy, significantly reducing its therapeutic efficacy	[112]

Metal-organic framework (MOF)	MOF		All types of cancer	Prussian blue MOF with NIR-II dye offers a hypoxia-modulating nanoplatform, suppressing HSP70 and enhancing tumour theranostics via NIR-II imaging	HSP70 suppression by the nanoenzyme platform alleviates tumour hypoxia and inhibits HSP70 expression, enhancing effectiveness in cancer theranostics	Without HSP70, the Prussian blue MOF with NIR-II dye would be less effective in modulating tumour hypoxia, leading to diminished suppression of HSP70 and a reduction in the overall effectiveness of the cancer theranostic platform. This would compromise both NIR-II imaging and therapeutic outcomes	[131]

**Table 2:** Clinical trials on function of heat shock protein in cancer.

Study title	Condition	Intervention	Results/outcomes	Status	Clinical trials identifier/reference
Vaccine therapy in preventing cervical cancer in patients with cervical intraepithelial neoplasia	Cervical cancer	Biological: pNGVL4a-Sig/E7(detox)/HSP70 DNA vaccine	HPV16 DNA vaccine for CIN2/3: Lesion regression, weak immune response. Heterologous prime-boost regimens may enhance efficacy; HSP70-based immunotherapy promising	Complete	NCT00054041
	Precancerous condition				

17AAG to treat kidney tumours in Von Hippel–Lindau disease	Hippel-Lindau disease	Drug: 17 allylamino-17-demethoxygeldanamycin drug: 18 FDG (Fludeoxyglucose 18F)	VHL gene ID improves clear cell RCC management. pVHL's angiogenic role explored. Further genotype–phenotype studies needed. HSP70 modulation in VHL patients with 17 AAG assessed	Complete	NCT00088374
	Kidney cancer				

SARC023: Ganetespib and sirolimus in patients with MPNST (malignant peripheral nerve sheath tumours)	Malignant peripheral nerve sheath tumours (MPNST)	Drug: Ganetespib	Ganetespib–sirolimus combo in MPNST had limited activity. Variable HSP70 changes suggest suboptimal Ganetespib levels	Complete	NCT02008877
	Sarcoma	Drug: Sirolimus			

HSP 70 to quantify and characterise circulating tumour cells (HSP70CTC)	Melanoma (stage IV)	Diagnostic test: CTC isolation by HSP70	Research on HSP70 for quantifying circulating tumour cells (CTCs) in advanced tumours, comparing it to EpCAM method, correlating molecular alterations and imaging parameters	Ongoing	NCT04628806
	Sarcoma				
	Squamous cell carcinoma				
	Pancreatic cancer (stage IV)				
	Prostate cancer				
	Breast cancer (stage IV)				

Pilot study with the aim to quantify a stress protein in the blood and in the urine for the monitoring and early diagnosis of malignant solid tumours (EXODIAG)	Cancer	Blood samples	Pilot study on HSP70 exosomes as a solid cancer biomarker, detecting and quantifying them in the blood of breast, lung and ovarian cancer patients	Ongoing	NCT02662621
		Urine samples			

RNA and heat shock protein biomarkers in radiation-induced fibrosis in breast cancer (SPLICI-Rad)	Breast carcinoma	Skin biopsies	Study comparing skin fibroblast reactions to radiation in two patient groups with different radiosensitivity, emphasising HSP70's role in predicting radiation-induced fibrosis	Ongoing	NCT03000764
	Fibrosis	Blood samples			

Relationship between the polymorphism of HSP70 gene and hepatocellular carcinoma	Hepatocellular carcinoma	Genetic: Polymorphisms of HSP70 and TNF promoter	HSP70's role in hepatocellular carcinoma (HCC) and its impact on early hepatocarcinogenesis and antitumour immunity, with a focus on the clinical relevance of TNF-α promoter polymorphism	Ongoing	NCT00769535

Heat shock protein 70 to quantify and characterise the circulating tumour cells (HSP70CTC)	Melanoma stage IV	Diagnostic test done: CTC isolation by HSP70	Uses HSP70 for CTC quantification in advanced tumours, comparing with EpCAM pre/post-treatment, correlating molecular alterations and imaging	Ongoing	NCT04628806
	Sarcoma				
	Squamous cell carcinoma				
	Pancreatic cancer stage IV				
	Prostate cancer				
	Breast cancer stage IV				

Study using vaccination with HSP70 for the treatment of CML in chronic phase	Leukaemia, Myeloid, chronic	Biological: HSP70	Study uses tumour-derived HSP70 for cancer immunisation, evaluating antitumour immunity and cytogenetic remission, guiding combined cytostatic therapy and HSP70 modulation trials	Complete	NCT00027144
	Leukaemia, myeloid, Philadelphia-positive				

**Table 3:** Patents related to HSP70 for cancer treatment and diagnostic applications.

Patent title	Claim	Patent no.	Patent filing date	Applicant	Reference
Methods for screening anticancer drugs inhibiting interactions between AIMP2-DX2 and HSP70	This patent targets anticancer agents disrupting AIMP2-DX2 and HSP70 binding, reducing AIMP2-DX2 levels for cancer therapy	KR102297505B1	2016-03-07	Centre for BioConvergence, South Korea	[219]
Methods of treating cancer by administering low levels of heat-shock protein 70	This patent proposes cancer treatment with HSP70, administered at 0.0003–3 mg/day through different routes, targeting various cancers, solely using HSP70 without additional therapies or complexing agents	WO2013103792A2	2013-01-04	Beach tree Labs, incorporated	[220]
Small-molecule HSP70 inhibitors	Patent targets HSP70/DnaK through a novel scaffold, inhibiting them to treat neoplastic diseases and reduce cancer cell mitochondrial HSP70 levels. Includes screening methods for inhibitors using the pocket's structure	US20170014434A1	2015-02-26	University of Pennsylvania Penn, Wistar Institute of Anatomy and biology, The United States	[221]
Methods for heat shock protein dependent cancer treatment	This invention treats HSP70-dependent cancers by exposing cancer cells to a low concentration of a dihydropyrimidinone compound and a proteasome inhibitor, resulting in a synergistic effect	US8754094B2	2008-08-15	Research foundation of state University of New York, University of Pittsburgh	[222]
Therapeutic and diagnostic anti-HSP70 antibodies	Patent focuses on using specific antibodies to target HSP70's extracellular epitope in cancer, particularly sarcomas and carcinomas	US8440188B2	2011-04-06	Multimmune GmbH	[223]
Quantitative assay for HSP70 protein in body fluids	This invention treats HSP70-dependent cancers by combining a dihydropyrimidinone compound and a proteasome inhibitor at low concentrations, producing a synergistic effect on cancer cells	US11460472B2	2016-01-27	Multimmune GmbH	[224]
HSP70 based combination therapy	Patent suggests combining HSP70-based pharmaceuticals with immunotherapeutic agents for treating tumours and infectious diseases, emphasising HSP70's pivotal role in the approach	US20200237860A1	2018-08-31	Multimmune GmbH	[225]
Biomarkers for breast tumours from HSP70-associated peptides	Invention identifies tumour-specific peptide biomarkers linked to HSP70 for breast cancer diagnosis and treatment, utilised in diagnostic assays and antitumour vaccine development	WO2013037714A1	2012-09-07	The college of the holy and undivided trinity of Queen Elizabeth, Dublin	[226]
Manipulation of HSP70 and IRE1Alpha protein interactions	Invention modifies HSP70-IRE1α interaction to enhance protein yield and treat diseases, including cancer, associated with abnormal apoptotic activity	US20130280269A1	2011-06-08	National University of Ireland	[227]
Anti-HSP70 antibodies and therapeutic uses thereof	The invention provides HSP70-specific antibodies for cancer treatment, enhancing HSP70 uptake by antigen-presenting cells in patients	WO2023056361A1	2022-09-29	The University of Texas system, Asylia therapeutics, The United States	[228]
Antigen-binding fusion proteins with modified HSP70 domains	The invention utilises fusion proteins with an antigen-binding domain and a modified HSP70 to induce immune responses for diseases tied to specific antigens, highlighting HSP70's immune modulation role	US11718683B2	2019-08-05	General Hospital Corp, Aperisys Inc	[229]
Monoclonal antibodies targeting HSP70 and therapeutic uses thereof	Agents targeting HSP70, including antibodies and chimaeric antigen receptors, are proposed for cancer treatment by boosting HSP70 uptake by antigen-presenting cells	US20230128075A1	2021-03-26	Asylia therapeutics, University of Texas system	[230]

## Data Availability

Data sharing was not applicable to this article as no datasets were generated or analysed during the current study.
